# ALM Resuscitation Without Transfusion Improves Platelet Function and Survival After Liver Injury and Uncontrolled Hemorrhage

**DOI:** 10.3390/medicina62030453

**Published:** 2026-02-27

**Authors:** Hayley Letson, Geoffrey Dobson

**Affiliations:** Heart, Sepsis and Trauma Research Laboratory, College of Medicine and Dentistry, James Cook University, Townsville, QLD 4811, Australia; geoffrey.dobson@jcu.edu.au

**Keywords:** platelets, fresh whole blood, fresh frozen plasma, transfusion, trauma, non-compressible hemorrhage, resuscitation, ALM

## Abstract

*Background and Objectives*: Traumatic hemorrhage is a leading cause of death. Our aim was to examine the effect of adenosine, lidocaine and magnesium (ALM) resuscitation therapy with and without fresh frozen plasma (FFP) or fresh whole blood (FWB) in a rat model of non-compressible hemorrhage. *Materials and Methods*: Anesthetized adult male Sprague-Dawley rats (439 ± 46 g) randomly assigned to (1) Shams (surgical trauma and liver isolation only without hemorrhage) (n = 34), (2) Saline controls (n = 34), or (3) ALM therapy (n = 34), underwent liver resection and uncontrolled bleeding. After 5 h 3% NaCl ± ALM bolus and 0.9% NaCl ± ALM drip fluid resuscitation, each group was randomized to receive no transfusion (NT) (n = 10 per treatment group), FFP (n = 12), or FWB (n = 12), and monitored for 72 h. Survival, hemodynamics, lactate, hematology, coagulation, platelet function, and lung histopathology were measured. *Results*: Sham, Saline and ALM NT survival were 50%, 0% and 100%. Sham survival increased to 75% with FFP, but not FWB (50%), and only marginally in the Saline group (8% and 17%, respectively). ALM protection was lost after 1–2 days with FFP and FWB (8% and 0% survival). Mortality was associated with acute lung injury, inflammation, activation of innate immunity, intrinsic hypocoagulopathy, and metabolic acidosis. Survival was associated with maintained platelet count and aggregation. Acute phase protein fibrinogen increased ~2.5 times in both survivors and non-survivors. *Conclusions*: ALM therapy without FFP or FWB transfusion significantly improved survival, reduced lung injury, preserved platelet function, and decreased immune and metabolic dysfunction. Blood products administered 5 h after injury did not significantly improve survival after non-compressible hemorrhage. Surgical trauma (laparotomy and liver isolation) also contributed to poor outcomes. The trauma and transfusion-related multi-system failure requires further investigation.

## 1. Introduction

Traumatic hemorrhagic shock is a leading cause of potentially preventable mortality, particularly in far-forward battlefield environments and prehospital civilian environments [1,2,3]. Volume resuscitation at the point-of-injury is essential to raise mean arterial pressure (MAP) and ensure adequate oxygen delivery to tissues [4]. Fresh whole blood (FWB) was the primary resuscitation fluid during World Wars I and II and the Korean War until the Vietnam War, with a wider adoption of crystalloids and colloids [5]. However, the use of high fluid volumes became associated with hemodynamic compromise, trauma-induced coagulopathy, acidosis and “shock lung” [6,7]. In recent years, crystalloids have mostly been replaced with component therapy (1:1:1 plasma:platelets:red blood cells) [4,5,8]. Two randomized clinical trials have also shown a survival benefit with fresh frozen plasma (FFP), although its use remains controversial [9,10]. In addition, there are logistical problems using FFP far-forward because it requires special handling, storage and preparative requirements compared to whole blood, which can be obtained from ‘buddy’ donors in the field [6,11]. Fresh whole blood has other physiological advantages, and two retrospective trials have shown a potential survival benefit of FWB or equivalence to component therapy in severe combat casualties [12,13]. Despite this, use of prehospital transfusion remains rare and widespread adoption faces ongoing challenges, particularly in rural and remote areas, which are disadvantaged by limited resources and availability of medical support [14,15].

We have been developing a small-volume adenosine, lidocaine and magnesium (ALM) fluid therapy for point-of-injury and prolonged field care after hemorrhagic shock [16,17]. ALM bolus and drip resuscitation therapy led to 3-day survival in the rat model of non-compressible hemorrhage, compared to 22 h for Saline controls [18]. The ALM survival phenotype was associated with improved cardiac function, correction of coagulopathy, platelet preservation, reduced inflammation, and upregulation of the master genes of metabolism and mitochondrial function (Ampk, Sirt-1, Pgc1a, Mtco3, and Tfam) in central tissues [18,19,20,21]. The aim of the present study was to examine the effect of FFP or FWB transfusion in addition to ALM therapy in the rat model of laparotomy, liver resection and non-compressible hemorrhage.

## 2. Materials and Methods

### 2.1. Animals and Ethics

**Figure 1:**
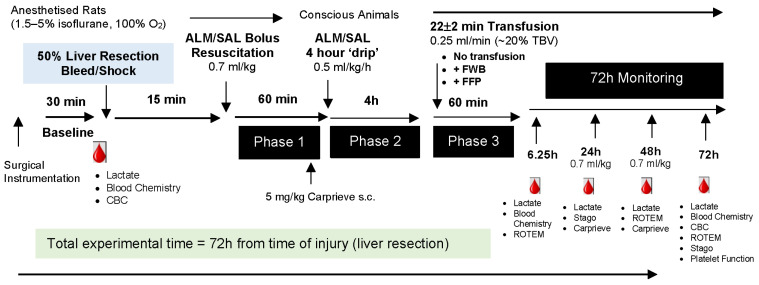
Schematic of the Study Protocol. Animals had 30 min baseline stabilization period after surgical instrumentation prior to laparotomy, liver resection, and uncontrolled bleeding. Phase 1 resuscitation commenced 15 min after liver resection, and animals received a 0.7 mL/kg bolus 3% NaCl ± ALM. Sixty minutes after bolus administration, Phase 2 resuscitation of 0.9% NaCl ± ALM drip commenced and continued for 4 h. During Phase 3 transfusion, animals received either no transfusion, 20% TBV FFP, or 20% TBV FWB. Animals received an additional 0.7 mL/kg 0.9% NaCl ± ALM bolus 24 h and 48 h after liver resection. Sham animals underwent surgical instrumentation, laparotomy and liver isolation without resection and bleeding and received the same Phase 1 and 2 treatment as Saline controls (3% NaCl bolus/0.9% NaCl drip). Animals received 5 mg/kg Carprieve^®^ s.c. prior to recovery from anesthesia and at 24 h and 48 h after liver injury. ALM, adenosine, lidocaine, magnesium; CBC, complete blood count; FFP, fresh frozen plasma; FWB, fresh whole blood; ROTEM, rotational thromboelastometry; SAL, saline; TBV, total blood volume.

This study was approved by James Cook University Animal Ethics Committee (A2296) and US Army Animal Care and Use Review Office (ACURO: SO150053) and complies with the *Australian Code for the Care and Use of Animals for Scientific Purposes* (8th Edition, 2013) and National Institutes of Health *Guide for Care and Use of Laboratory Animals* (8th Edition, 2011). The study is reported in accordance with the Animal Research: Reporting In Vivo Experiments (ARRIVE) guidelines [22] (Supplementary Material S1). Conventional adult male Sprague-Dawley rats (n = 102; 439 ± 46 g) sourced from the non-specific pathogen-free James Cook University breeding colony, were housed in individually ventilated Tecniplast cages in a 14–10 h dark–light cycle with free access to environmental enrichment, food and water ad libitum. Animals were randomly assigned using a random number generator (GraphPad Prism 8; GraphPad Software L.L.C, San Diego, CA, USA) to (1) Sham No Transfusion (NT) (n = 10), (2) Saline NT (n = 10), (3) ALM NT (n = 10), (4) Sham FFP (n = 12), (5) Saline FFP (n = 12), (6) ALM FFP (n = 12), (7) Sham FWB (n = 12), (8) Saline FWB (n = 12), or (9) ALM FWB (n = 12). Animals were anesthetized with isoflurane 1.5–5% in 100% oxygen during surgical instrumentation, laparotomy, liver resection, and throughout Phase 1 bolus resuscitation. Carprieve^®^ (Norbrook Laboratories; Newry, Northern Ireland) (carprofen 5 mg/kg s.c.) was administered prior to recovery from anesthesia for postoperative analgesia and again at 24 h and 48 h (Figure 1).

### 2.2. Surgical Protocol

Anesthetized animals had sterile chronic catheters (Access Technologies, Skokie, IL, USA) implanted in the left femoral artery and vein for blood pressure monitoring (BridgeAMP coupled to Powerlab; ADInstruments, Bella Vista, Australia), blood sampling, and fluid/drug infusions respectively. Lead II electrocardiogram was subcutaneously implanted for heart rate monitoring (BioAMP/Powerlab). Animals had a 30 min baseline stabilization period after surgical instrumentation, with the exclusion of any animal that exhibited complex ventricular arrhythmias or a sustained fall in MAP < 80 mmHg (Figure 1).

### 2.3. Liver Injury and Uncontrolled Bleeding

Animals had a 3 cm transverse laparotomy, and 50% of the left lateral and medial liver lobes were resected with sharp dissection and allowed to bleed freely into the peritoneal cavity, as in previous studies [19,20]. This model of liver resection-induced non-compressible hemorrhage has been validated [23], and is more clinically relevant than other pressure- or volume-controlled hemorrhage models [24,25]. As the largest solid abdominal organ, the liver is susceptible to both blunt and penetrating injury, and subsequent non-compressible hemorrhagic shock [26,27]. Consistency of liver injury was ensured by comparing the weight of resected liver with total body weight (Saline NT: 0.52 ± 0.04%, ALM NT: 0.54 ± 0.05%, Saline FFP: 0.53 ± 0.04%, ALM FFP: 0.57 ± 0.06%, Saline FWB: 0.54 ± 0.07%, ALM FWB: 0.58 ± 0.05%; *p* = 0.117). Sham animals underwent surgical instrumentation, laparotomy and liver isolation, without resection and bleeding, to determine surgical trauma-induced responses in the absence of traumatic hemorrhagic shock.

### 2.4. Resuscitation and Transfusion

Fifteen minutes after liver injury, animals received a 0.7 mL/kg intravenous (IV) bolus of 3% NaCl (Saline and Sham) or 3% NaCl ALM (ALM; 1 mM adenosine, 3 mM lidocaine, 2.5 mM MgSO_4_; Sigma-Aldrich. Castle Hill, Australia [19]) via the femoral vein catheter (Figure 1). Animals were recovered from anesthesia after 60 min Phase 1 bolus resuscitation and received a 4 h infusion of 0.5 mL/kg/h 0.9% NaCl (Saline and Sham) or 0.9% NaCl ALM (ALM; 0.25 mg/kg/h adenosine, 0.5 mg/kg/h lidocaine, 0.25 mg/kg/h MgSO_4_ [19]) (Figure 1). After Phase 2 drip resuscitation was complete, animals received NT, FFP or FWB according to group allocation for Phase 3 transfusion. The timing of resuscitation and transfusion was chosen to reflect prehospital times for trauma patients in regional, rural and remote Australia, which are significantly prolonged (4–9 h) [28,29] compared to those in the United States [14,30] and Europe [31], which average 50–60 min. Northern Australia currently has very limited capability for prehospital transfusion due to availability and logistic challenges, with transfusion commencing on hospital admission, often hours after injury. FFP and FWB, equivalent to 20% total blood volume calculated from [(0.06 × weight) + 0.77] [32], warmed to 37 °C, were transfused at 0.25 mL/min through a Hemo-Nate Syringe Filter (Sound Veterinary Equipment; Rowville, Australia). Mean transfusion time was 21.7 ± 2.2 min. Animals were monitored for 72 h post-injury with administration of 0.7 mL/kg IV bolus of 0.9% NaCl (Saline and Sham) or 0.9% NaCl ALM (ALM; 1 mM adenosine, 3 mM lidocaine, 2.5 mM MgSO_4_) at 24 h and 48 h (Figure 1).

### 2.5. Preparation of FFP and FWB

FFP and FWB were prepared from donor syngeneic female Sprague-Dawley rats, matched according to breeding pair. Fresh shed citrated blood (FWB) collected from anesthetized donors was incubated at 37 °C with gentle rolling to maintain homogeneity until transfusion. For fresh frozen plasma (FFP), blood was left to sit at room temperature for 30 min, then passed through a neonatal leukocyte reduction filter (Nanodyne Filter 0.2 μm; Pall Corporation, Dandenong South, Australia) and centrifuged at 4000× *g* for 15 min [33]. Plasma was separated from red cells and stored at −80 °C until use.

### 2.6. Blood Sampling, Moribund Score and Mortality

Animals were continuously monitored for the first 6 h after liver injury and reconnected for hemodynamic monitoring every 12 h until 72 h. Blood was sampled at baseline (post-surgical instrumentation), end Phase 3 transfusion (6.25 h post-injury), and at 24 h, 48 h and 72 h for lactate, blood chemistry, complete blood count, and coagulation and platelet function assessments (Figure 1). Mortality was defined as MAP < 25 mmHg for 5 min. Animals were assessed twice daily using the humane endpoints of Morton [34], Toth [35], and Nemzek and colleagues [36]. Weight loss, behavior/alertness, mobility, appearance/coat condition, wound healing, breathing/respiration, food/water intake and feces were scored 0–3. Any animal scoring ≥10, or 3 in any category, was categorized as being in a moribund state preceding imminent death and was euthanized. Surviving animals were sacrificed at the end of the 72 h experimental period. At the time of sacrifice, animals were re-anesthetized with isoflurane for blood sampling from the femoral artery catheter, followed by euthanasia with 100 mg/kg IV pentobarbitone sodium (Lethabarb^®^; Virbac Australia; Milperra, Australia).

### 2.7. Blood Chemistry, Hematology, Coagulation and Platelet Function

Blood chemistry and lactate were measured using a Radiometer ABL800 analyzer (Radiometer Pacific; Mount Waverley, Australia), and VetScan HM5 (Zoetis Diagnostics; Rhodes, Australia) was used for complete blood counts, as in previous studies [18,20].

Prothrombin time (PT, s), activated partial thromboplastin time (aPTT, s), fibrinogen (g/dL), tissue activatable fibrinolysis inhibitor (TAFI, %), Protein C (%), and antiplasmin (%) were measured in citrated plasma on the STA Compact (Diagnostica Stago; Doncaster, Australia).

Rotational thromboelastometry (ROTEM^®^; Tem International, Munich, Germany) was conducted according to the manufacturer’s instructions and previous studies [20,37]. Whole blood collected in 3.2% sodium citrate tubes was warmed to 37 °C. Three assays were performed: EXTEM (extrinsically activated test using tissue factor), INTEM (intrinsically activated test using ellagic acid), and FIBTEM (fibrin-based EXTEM-activated test with 50 μg/mL cytochalasin D, to inhibit platelet contribution to clot formation).

Platelet function was assessed in platelet-rich plasma (PRP) using the PAP-8e Platelet Aggregation Profiler (BioData Corporation; Horsham, PA, USA) and agonists ADP (200 μM) and Collagen (equine tendon, 100 μg/mL) from Helena Laboratories (Mount Waverley, Australia). PRP was prepared using a standardized technique of double centrifugation [38].

### 2.8. Lung Histopathology

Sections of lung collected at the time of sacrifice were fixed in 10% neutral buffered formalin, processed, and paraffin-embedded. Six 4 μm paraffin-embedded sections were cut from each block, spaced at 100 μm intervals and spanning the entire tissue sample. Alternate sections (2nd, 4th and 6th sections) were stained with hematoxylin and eosin (H&E). Stained sections were visualized with light microscopy (Nikon Eclipse i50; Nikon, Tokyo, Japan) and digitized (Nikon NIS-Elements Basic Research Software) for semi-quantitative assessment of pathological changes. Lungs were examined for vacuolation, alveolar infiltration, inflammation, hemorrhage/congestion, and epithelial degeneration. Sections were scored from 0 to 4 (none-severe, >80% tissue affected) for each parameter by two blinded investigators (total possible score = 20).

### 2.9. Statistical Analysis

A priori power analysis was conducted using the G-power^3^ program (Heinrich Heine University Düsseldorf; Düsseldorf, Germany) to determine the minimum sample size to minimize Type 1 errors (outcome measure = MAP 6.25 h post-injury [19]; effect size = 1.33; α err prob = 0.05; Power (1-β error prob) = 0.8; calculated sample size n = 10). SPSS Statistical Package 25 was used for all statistical analyses (IBM, Sydney, Australia). Survival was assessed using the Kaplan–Meier method with a log-rank test for comparison between groups. Data normality was assessed graphically and with the Shapiro–Wilk test. Data are expressed as mean ± standard deviation (SD). Normally distributed data were analyzed using one-way analysis of variance (ANOVA). Longitudinal data were assessed with General Linear Model Repeated Measures ANOVA. Within-group differences were assessed using a paired-samples *t*-test. Non-parametric data were analyzed using a Kruskal–Wallis test followed by Dunn’s test. Point-biserial correlations were conducted to determine the relationship between continuous variables and mortality. Statistical significance was defined as *p* < 0.05.

## 3. Results

### 3.1. Survival

**Figure 2:**
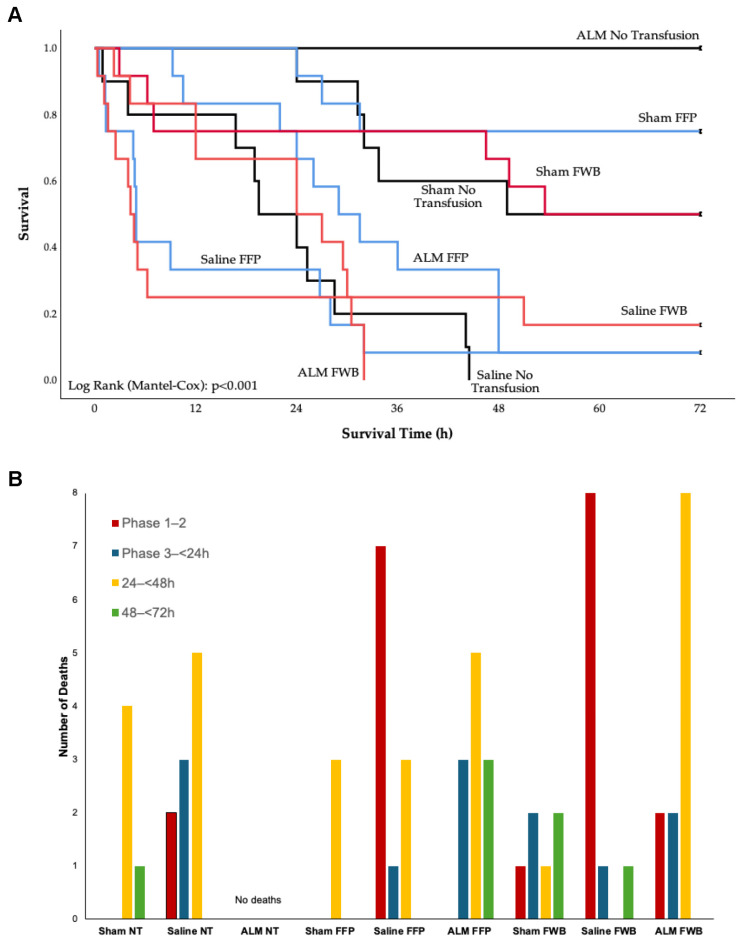
(**A**) Kaplan–Meier survival curve for Shams, Saline controls and ALM treatment group receiving no transfusion (black), FFP (blue), or FWB (red) following laparotomy, liver resection and uncontrolled hemorrhage. Log Rank (Mantel Cox): χ^2^ = 59.715, df = 8, *p* < 0.001. (**B**) Number of deaths per study phase. ALM, adenosine, lidocaine, magnesium; NT, no transfusion; FFP, fresh frozen plasma; FWB, fresh whole blood.

Survival in Sham, Saline and ALM NT groups was 50%, 0% and 100%. Saline NT had significantly reduced mean survival time (23 h) compared to Sham (53 h) and ALM (72 h) (*p* < 0.05) (Table 1, Figure 2). FFP increased Sham survival to 75%, with no change after FWB (50%). FFP and FWB increased overall survival in Saline controls from 0% to 8% and 17%, respectively, but did not increase survival time (16 h and 19 h). In contrast to 100% survival to 72 h in the ALM NT group, ALM FFP and ALM FWB had significantly reduced survival (8%, 34 h and 0%, 22 h, respectively) (*p* < 0.05) (Table 1, Figure 2).

### 3.2. Hemodynamics

Liver resection and uncontrolled bleeding led to a marked ~50% fall in MAP (*p* < 0.05 vs. Sham groups), which recovered at 15 min Phase 1 with a 0.7 mL/kg bolus of 3% NaCl or 3% NaCl ALM (Supplementary Figure S1A). There were no significant differences in heart rate (HR) between groups during Phases 1–3, or in survivors and non-survivors during the 72 h monitoring period (Supplementary Figure S1B). MAP and HR increased after Phase 1 when animals were recovered from anesthesia, and there was a trend towards higher HR at 36 h and 60 h (evening measurements) compared to 48 h and 72 h (Supplementary Figure S1A,B).

### 3.3. Blood Lactate and Chemistry

Saline NT and FFP animals, and all FWB animals that died early (Phase 1–2), were profoundly acidotic with blood pH ranging from 6.7 to 7.14, and high lactates (7–20 mM) (Table 2). In contrast to other groups that became moribund from Phase 3 to 24 h, which had significantly increased lactates and reduced pH, ALM FWB non-survivors during the same period had no change in blood pH (7.44) or lactate (1.65 mM). After 24 h, pH returned to 7.3–7.6 in non-survivors (except Sham FFP), but lactates remained elevated. In addition to prolonged acidemia, Sham FFP survivors had increased lactate at 72 h compared to all other groups (3.76 vs. <1.8 mM) (Table 2).

Survivors also had blood K^+^ levels comparable to baseline (~4.5 mM), whereas K^+^ increased up to 14.25 mM in moribund animals (Supplementary Table S1). Blood Na^+^ and Cl^−^ increased in earlier moribund groups (Phase 1–2), and after transfusion (Phase 3) in Sham and Saline FFP and FWB groups, but not ALM FFP and FWB. ALM FFP and FWB animals that died after 24 h also maintained normal base excess and bicarbonate, compared to other moribund groups, which showed marked falls (Supplementary Table S1).

### 3.4. Red Cell and Platelet Profile

Survivors at 72 h had significant 30–37% decreases in red blood cell (RBC) numbers and hematocrit (Hct), and 29–39% falls in hemoglobin (Hb) (Table 3). Non-survivors had similar percentage falls of up to 30% in RBC, Hb and Hct, except for ALM FWB, which only dropped 5–10%. At 72 h, platelet numbers in Sham NT, FFP and FWB survivors fell by 53%, 36% and 53% from baseline, respectively (Table 3). There was a significant negative correlation between platelet count from Phase 1 to 24 h and survival time (r*_pb_* = −0.451; *p* = 0.010). Sham and Saline animals that became moribund after 24 h had 54–95% and 80–94% falls in platelet numbers, respectively. Total platelets fell in ALM FFP and ALM FWB non-survivors at 24–48 h by 16% and 35% respectively, whereas platelet count in ALM NT survivors was only 7% lower than baseline (Table 3).

### 3.5. White Blood Cell (WBC) Profile

Total white cell count at 72 h in ALM NT group with 100% survival was comparable to baseline (10.68 × 10^9^/L vs. 12.19 × 10^9^/L), compared to a 25% decrease in WBCs in Sham NT survivors (Table 4). Transfusion of FWB in Shams led to a more profound leukopenia at 72 h (48% fall), whereas FFP preserved WBC in Shams (11% increase vs. baseline at 72 h). White cells fell significantly in all FFP and FWB moribund groups between 24 and 72 h (<2.85 × 10^9^/L), indicating immunosuppression; however, in contrast to the Saline NT group, which showed an exponential early decrease in WBC, FFP or FWB appeared to defend against these large falls in the first 48 h (Table 4).

Similar to WBC, Sham survivors also had 33–67% falls in lymphocytes, and 38–45% falls in monocytes, with the exception of Sham FFP animals, where circulating monocytes increased 1.35-fold at 72 h (Table 4). In the Saline and ALM groups, lymphocytes fell significantly in both survivors and non-survivors. Monocytes also decreased in Saline and ALM moribund groups (Table 4). Neutrophils increased 1.3 to 2.2-fold from baseline to 72 h in all survivors; however, there was no evidence of neutrophilia in moribund groups after 24 h. The neutrophil/lymphocyte ratio was increased in both survivors and moribund groups, including 4- to 16-fold increases in Sham survivors, 3.4- to 6-fold and 4- to 20-fold in Saline survivors and non-survivors, and 5.2-fold and 2.9- to 5.4-fold in ALM survivors and non-survivors, with no differences between NT, FFP, and FWB (Table 4). The monocyte/neutrophil ratio at 72 h fell 48–70% in Shams, and 80–86% in Saline FFP and FWB, compared to only 25% in ALM NT with 100% survival.

### 3.6. Coagulation

PT remained relatively constant except for the early FWB deaths (Phase 1–24 h), where it increased 3- to 8-fold (Table 5). In contrast, aPTT in all NT groups increased up to 2.6-fold, and similar prolongation was found after FFP and FWB transfusion, indicating hypocoagulopathy, which was not corrected until 72 h. These results were very similar to the small changes in EXTEM clot time (CT) and increases in INTEM CT in non-survivors (Table 5 and Table 6). In contrast to the correction of aPTT in survivors, the INTEM ROTEM equivalent showed increases at 72 h, indicating persistent hypocoagulopathy. However, maximum clot firmness (MCF) and maximum lysis (ML) indicated that the clots were stable with little hyperfibrinolysis (Table 6). Sham NT had a 3.5-times prolongation of INTEM CT, which was reduced by ~50% after FFP (1.24-times) or FWB (1.6-times).

Fibrinogen fell significantly by ~70% in early deaths to 24 h, but increased 2.5 times in 72 h survivors (*p* < 0.05 vs. baseline), which was consistent with FIBTEM results (Table 5 and Table 6). The increase occurred in the first 24 h and appears to remain high for 3 days. Similar to fibrinogen, TAFI levels increased up to 2-fold in survivors at 72 h (Table 5). In early moribund groups (Phase 1–24 h), no change in TAFI occurred with the exception of ALM FWB, which fell by 50% (*p* < 0.05 vs. ALM NT and FFP). In contrast, TAFI increased by 1.3 to 1.5-fold in Sham FWB and Saline FWB over the same period (*p* < 0.05 vs. ALM FWB), and almost doubled in ALM NT group to 112% at 24 h and 119% at 72 h. Protein C in early moribund groups (Phase 1–24 h) for Saline NT and ALM FWB underwent no change, whereas Saline FWB and all FFP groups increased 3- to 6-fold to 48 h (Table 5). Transfusion of FWB significantly reduced Protein C levels at 24–72 h with falls of 57%, 67% and 87% in Sham FWB, Saline FWB, and ALM FWB, respectively. Early deaths (Phase 1–24 h) were associated with reduced antiplasmin, except ALM FWB, which was comparable to baseline. Antiplasmin fell by 35% in the Sham NT group and did not recover, but increased by 15% and 37% in Sham FFP at 24 h and 72 h, respectively (Table 5). Antiplasmin also increased 1.5 to 1.8-fold in ALM NT and Sham FWB survivors.

### 3.7. Platelet Function

**Figure 3:**
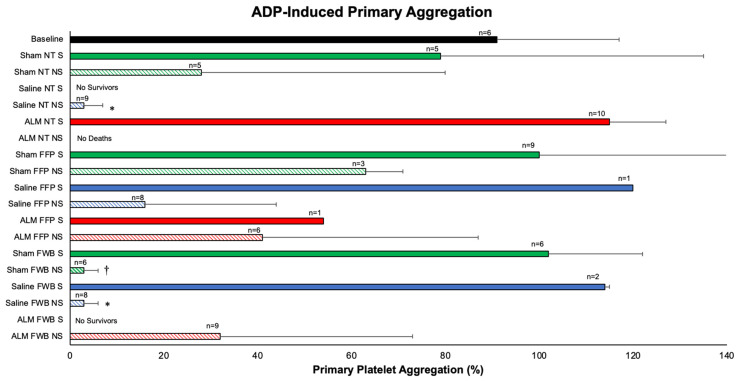
ADP-induced primary platelet aggregation in Shams, Saline controls and ALM treatment group survivors vs. non-survivors receiving no transfusion, FFP, or FWB after laparotomy, liver resection and uncontrolled hemorrhage. Samples were taken 72 h following liver resection (survivors, S) or at time of sacrifice in moribund animals (non-survivors, NS) (refer Section 2.6). Survivors indicated with solid fill and non-survivors indicated with diagonal pattern. * *p* < 0.05 compared to ALM NT survivors, Sham FFP survivors, and Sham FWB survivors; ^†^ *p* < 0.05 compared to ALM NT survivors. ADP, adenosine disphosphate; ALM, adenosine, lidocaine, magnesium; NT, no transfusion; FFP, fresh frozen plasma; FWB, fresh whole blood; S, survivors; NS, non-survivors.

ADP-induced primary platelet aggregation (ADP PA), defined as the extent of aggregation during the first minute, for Sham NT survivors was 13% lower than baseline (79 vs. 91%), whereas Sham NT non-survivors fell by 69% (Figure 3). Platelet aggregation was restored in Sham FFP and FWB survivors (1.1-times baseline), but fell by 31% and 93% in Sham FFP and FWB non-survivors, respectively. Similarly, the addition of FFP or FWB in Saline survivors restored platelet aggregation to ~1.3-times baseline, compared to falls of 83–93% in non-survivors (*p* < 0.05 vs. ALM NT). ALM NT aggregation was 1.27 × baseline, and all survived, whereas aggregation in ALM FFP and FWB non-survivors were 45% and 35% of baseline (Figure 3). ADP PA was very strongly correlated with survival (r*_pb_* = 0.857; *p* < 0.0001) and strongly correlated with survival time (r*_pb_* = 0.766; *p* < 0.0001). Collagen-induced platelet aggregation showed similar findings, including strong positive associations with survival (r*_pb_* = 0.667; *p* < 0.0001) and survival time (r*_pb_* = 0.627; *p* < 0.0001).

### 3.8. Lung Pathology

**Figure 4:**
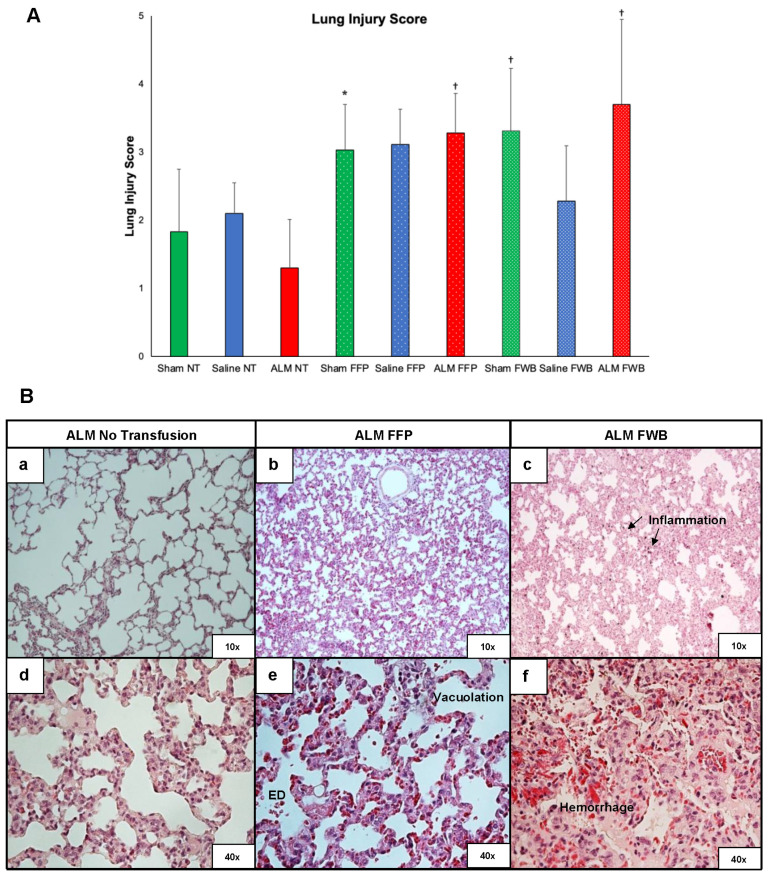
(**A**) Lung histology injury scores for Shams, Saline controls and ALM treatment group receiving no transfusion, FFP, or FWB after laparotomy, liver resection and uncontrolled hemorrhage. Data represent mean ± SD. * *p* < 0.05 compared to Saline NT and ALM NT; ^†^ *p* < 0.05 compared to all NT groups. (**B**) Representative histopathology images from ALM NT (a,d), ALM FFP (b,e), and ALM FWB group (c,f) at 10× and 40× magnification, showing evidence of inflammation, hemorrhage, cytoplasmic vacuolation, and epithelial degeneration (ED) in FFP and FWB groups. ALM, adenosine, lidocaine, magnesium; NT, no transfusion; FFP, fresh frozen plasma; FWB, fresh whole blood.

Total lung injury scores were significantly higher in Sham FWB, Saline FFP, ALM FFP, and ALM FWB compared to all NT groups (Figure 4A). Lung injury score showed a weak significant correlation with mortality (r*_pb_* = 0.296; *p* < 0.012), while hemorrhage/congestion score was moderately associated with death (r*_pb_* = 0.330; *p* < 0.005). ALM FFP had significantly increased hemorrhage and inflammation compared to ALM NT, and all ALM FWB animals showed mild-to-moderate hemorrhage and epithelial degeneration (*p* < 0.05 vs. ALM NT) (Figure 4B). ALM FWB animals had a distinct gross pathophysiological profile of lung congestion, edema, and excess pericardial fluid. No such lesions or congestion were found in the ALM NT group.

## 4. Discussion

Uncontrolled hemorrhage remains a leading cause of potentially survivable death in prehospital civilian and military environments [1,2,3]. We have previously shown that small-volume ALM therapy can resuscitate, correct trauma-induced coagulopathy, and provide multiple organ protection, in both small and large animal models of traumatic hemorrhage [17,18,19,20,21,39]. We report in the rat model of laparotomy, liver resection and uncontrolled hemorrhage, that transfusion of 20% total blood volume of FFP or FWB 5 h after resuscitation with ALM therapy reduced 72 h survival. In contrast, ALM without transfusion led to 100% survival. Mortality appeared to be related to transfusion-related acute lung injury (TRALI), reduced platelet function, metabolic and immune dysfunction, and hypocoagulopathy. We also found that surgical and abdominal trauma from laparotomy and liver isolation was a major contributor to poor outcomes because Shams (no hemorrhage) had similar profiles. Higher systemic fibrinogen levels in survivors and non-survivors appeared to be due to activation of the innate immune response induced by surgical trauma. These results are discussed next.

### 4.1. Sham Mortality Was Due to the Trauma of Surgery, Not Hemorrhage

An unexpected result was that 50% of Sham animals that did not receive a transfusion died, which was slightly improved after FFP infusion (75% survival), but not with FWB. Shams underwent anesthesia, surgical catheterization involving groin and neck cut-downs, a 3 cm open laparotomy, and liver isolation, but no resection. Our data emphasize the importance of including Shams to accurately interpret the physiological responses to resuscitation therapies in animal models. Unfortunately, many preclinical studies investigating blood product transfusion efficacy either do not include Shams or they are not present for the entire experiment [40,41,42], which may lead to contradictory results in the literature.

### 4.2. ALM Therapy Led to 100% Survival Without Blood Products

The other unexpected finding of our study was that transfusion of FFP or FWB 5 h after ALM resuscitation resulted in higher mortality than ALM alone, which led to 100% survival after 3 days. Possible reasons include the timing of transfusion, currently unknown contraindications, as well as the effect of these blood products on lung pathology and immune function, and are discussed below. Similarly, FFP or FWB failed to improve survival in Saline controls, although the sample sizes were smaller due to high early mortality prior to the transfusion phase. The limited resuscitative and hemostatic capacity of saline alone is supported by previous studies [37,43,44].

### 4.3. FWB and FFP Led to Poor Outcomes in All Groups

Mortality in FFP and FWB groups appeared to be associated with TRALI, which was not apparent in groups without transfusion. In addition, lung hemorrhage was not present in the Sham group that did not receive blood products, confirming that this effect was directly related to transfusion. Mortality in ALM animals receiving FWB or FFP was accompanied by pulmonary congestion and hemorrhage, edema, inflammation, and epithelial degeneration, which may be associated with varying degrees of early or delayed TRALI. The pathophysiology of TRALI is believed to arise from a breach of endothelial, interstitial, and epithelial barriers driven by inflammation and exacerbated by fluid therapies, leading to extravasation of proteinaceous fluid into the airspace [45], and similar to the pathology we observed. Our findings are consistent with studies reporting TRALI and acute respiratory distress syndrome (ARDS) following both FFP and FWB administration after hemorrhagic trauma [40,46].

### 4.4. Mortality, Platelet Exhaustion and Coagulopathy

In addition to transfusion, another important factor associated with mortality was a dramatic loss of ADP- and collagen-induced platelet aggregation. Our findings of a significant strong correlation between both ADP- and collagen-induced primary platelet aggregation are consistent with clinical studies reporting reduced platelet function in trauma patients, which was associated with increased mortality (up to 10-fold) [47,48]. An “exhausted platelet syndrome” begins with an initial hyperactivation of platelets from the widespread release of ADP from injured endothelial cells, which subsequently become unresponsive via mechanisms not well understood [49]. Loss of platelet aggregation may also be exacerbated by reduced platelet numbers. Since Sham NT non-survivors had 69% and 93% falls in platelet aggregation and count, respectively, we conclude that reduced platelet function was most likely due to surgical trauma [50], not the use of blood products or hemorrhage. This illustrates the complexity of our polytrauma model, on one hand, and yet its ability to tease apart the different mechanisms contributing to death after hemorrhagic shock, on the other.

Platelet exhaustion was also associated with hypocoagulopathy in all groups, as indicated by a prolonged aPTT and prolonged INTEM clot times, with some failing to form a clot. The use of FFP or FWB partially corrected hypocoagulopathy, with reduced INTEM clot times and improved INTEM and FIBTEM clot firmness. Stronger clots after administration of blood products are often supported by higher circulating fibrinogen levels, which is consistent with significantly higher FIBTEM MCF. However, increased fibrinogen normally favors platelet aggregation by binding to platelet GPIIb/IIIa receptors [51], yet this was not the case in our moribund animals with or without blood products. How do we explain this paradox? One possibility is that the 2- to 2.8-fold increase in systemic fibrinogen, irrespective of treatment, was part of the positive acute-phase response to inflammation, trauma, and/or tissue damage, separate from its effect in combining with platelets and forming strong clots [52]. Once present in blood, fibrinogen and other acute-phase proteins, such as C-reactive protein, C3 complement and alpha-globulin glycoprotein, can in turn modulate the inflammatory response via the recruitment of immune cells and cytokine production [52]. This may also be related to the leukopenia and lymphopenia we found over the 72 h experimental period, and may contribute to a trauma-induced immunosuppression. Thus, we conclude that viscoelastic INTEM and FIBTEM parameters in vitro do not necessarily imply that increased clot firmness is occurring in vivo, given impaired platelet aggregation, reduced platelet count, and 50% falls in platelet/fibrinogen ratios that are incompatible with the formation of viable clots. This linkage between acute-phase response to trauma, inflammation and coagulopathy is an area that requires further study.

### 4.5. Survivors Preserved Platelet Aggregation and Reduced Coagulopathy and Immune Activation

In contrast to non-survivors, platelet aggregation in survivors was maintained close to baseline, regardless of the use of blood products. Maintenance of platelet aggregation in survivors occurred despite ~50% falls in platelet numbers in most groups. Preserved platelet function was also associated with reduced coagulopathy among the survivors, including normal PT, aPTT, EXTEM and FIBTEM clot times, as well as a recovery of INTEM CT towards baseline. This is consistent with what is now known about the significant contribution platelet dysfunction makes to early trauma-induced coagulopathy (TIC) and the potential limitation of FFP alone [10].

Key to ALM therapy survival, in addition to preservation of platelet function, was attenuation of immune activation, reduced blood lactate and normal acid-base status. Immune dysfunction and hyperinflammation after traumatic injury and hemorrhage are important contributors to secondary injury progression, including immunosuppression, infection and development of multiple organ dysfunction, including lung injury [53]. Trauma-induced immune dysfunction and inflammation may, in turn, be exacerbated by blood product administration, an effect known as transfusion-associated immunomodulation (TRIM) [54]. It is possible in this study that delayed administration of FFP or FWB after successful resuscitation, correction of TIC and stabilization with ALM therapy led to a “second-hit” injury.

### 4.6. Clinical Significance and Limitations

While administration of fresh whole blood in humans appears to offer clinical advantages over balanced component therapy (red blood cells, plasma, and platelets) [55], the quality of the data is poor, and more high-quality studies are required [3,56]. In our rat model, transfusion with FWB or FFP 5 h after fluid resuscitation surprisingly led to poor outcomes, which may be a reflection of the severity of our model and timing of transfusion, with multiple clinical studies demonstrating benefit with earlier transfusion [57,58,59]. Our preclinical model combining uncontrolled hemorrhage with a laparotomy and liver manipulation had a high mortality and high individual variability, which limited time-matched comparisons of all endpoints. Despite this, the model may have military significance in Role 2 facilities where casualties with suspected abdominal hemorrhage requiring an emergent laparotomy have high mortality rates [60]. Our data showing that ALM therapy alone was effective against non-compressible hemorrhage and major surgical trauma with 100% survival is directly relevant to battlefield medicine and forward surgical facilities when blood products may be in short supply. In addition to the model’s severity, another limitation of our study was that the individual effects of FFP or FWB alone were not tested in our model. Finally, in order to comply with ethical restrictions and the 3Rs of preclinical studies, only male rats were evaluated, with female rats used exclusively as donors for blood products. Despite no current evidence in rats, clinical studies have shown that sex-mismatched transfusion is associated with increased mortality [61]. Further studies are required to determine any sex-specific differences, particularly relating to sex hormones and transfusion-induced immunoinflammatory responses.

## 5. Conclusions

In the rat model of laparotomy, liver resection, and hemorrhage, Sham groups had 25–50% mortality, indicating that the trauma of surgery was a significant contributor to poor outcomes. ALM resuscitation therapy without blood products led to 100% survival, and was associated with improved hemostasis, platelet aggregation and metabolic and immune function. Our study showed that ALM protection was lost with administration of FFP or FWB, which led to reduced survival, and was related to acute lung injury, platelet exhaustion, hypocoagulopathy, and transfusion-associated immunomodulation. Further studies are required to evaluate the underlying mechanisms and to assess the clinical significance of our findings.

## Figures and Tables

**Table 1:** Survival summary.

Time	Sham NT	Saline NT	ALM NT	Sham FFP	Saline FFP	ALM FFP	Sham FWB	Saline FWB	ALM FWB
72 h Survival (%)	5/10 (50%)	0/10 (0%)	10/10 (100%)	9/12 (75%)	1/12 (8%)	1/12 (8%)	6/12 (50%)	2/12 (17%)	0/12 (0%)
Survival Time (h)	53 ± 21	23 ± 14 *	72 ± 0	61 ± 20	16 ± 21 ^†^	34 ± 18 ^‡^	50 ± 28	19 ± 28 ^†^	22 ± 11 *
Phase 1–2	No deaths	2	No deaths	No deaths	7	No deaths	1	8	2
Phase 3–<24 h	No deaths	3	No deaths	No deaths	1	3	2	1	2
24–<48 h	4	5	No deaths	3	3	5	1	No deaths	8
48–<72 h	1	No survivors	No deaths	No deaths	No deaths	3	2	1	No survivors

Survival time presented as mean ± SD. ALM, adenosine, lidocaine, magnesium; NT, no transfusion; FFP, fresh frozen plasma; FWB, fresh whole blood. * *p* < 0.05 vs. Sham NT, Sham FFP, Sham FWB, and ALM NT; ^†^ *p* < 0.01 vs. Sham NT, Sham FFP, Sham FWB, and ALM NT; ^‡^ *p* < 0.05 compared to Sham FFP and ALM NT.

**Table 2:** Arterial pH and Lactate for Shams, Saline controls, and ALM group without transfusion, and with FFP or FWB following laparotomy, liver resection and hemorrhage.

Parameter (Reference Range)	Time	Sham NT	Saline NT	ALM NT	Sham FFP	Saline FFP	ALM FFP	Sham FWB	Saline FWB	ALM FWB
pH [7.366–7.462]	P1–P2	No deaths	NA	No deaths	No deaths	7.14 ± 0.24 ^7^	No deaths	6.72 ^1^	6.94 ± 0.22 ^8^	7.00 ± 0.05 ^2^
	P3	7.44 ± 0.04	7.45 ± 0.07 ^8^	7.47 ± 0.04	7.48 ± 0.03	7.39 ± 0.12 ^5^	7.40 ± 0.17	7.43 ± 0.07 ^11^	7.39 ± 1.0 ^4^	7.44 ± 0.04 ^10^
	P3–<24 h	No deaths	7.10 ^1^	No deaths	NA	7.30 ^1^	7.16 ± 0.27 ^3^	7.09 ± 0.42 ^2^	7.26 ^1^	7.44 ± 0.15 ^2^
	24–<48 h	7.49 ^1^	7.38 ± 0.18 ^4^	No deaths	7.21 ± 0.20 ^3^	7.32 ± 0.40 ^3^	7.44 ± 0.10 ^5^	7.46 ^1^	No deaths	7.43 ± 0.08 ^8^
	48–<72 h	No sample	No survivors	No deaths	NA	No deaths	7.35 ± 0.25 ^3^	7.42 ± 0.06 ^2^	7.44 ^1^	No survivors
	72 h	7.54 ± 0.03 ^5^**	No survivors	7.52 ± 0.07	7.54 ± 0.04 ^9†^	7.53 ^1^	7.45 ^1^	7.56 ± 0.04 ^6^	7.56 ± 0.0.4 ^2^	No survivors
Lactate (mM)	Baseline	1.11 ± 0.35	1.30 ± 0.43	1.37 ± 0.24	1.72 ± 0.41 ^††^	1.58 ± 0.30	1.62 ± 0.21	1.30 ± 0.32	1.28 ± 0.38	1.20 ± 0.27
	P1–P2	No deaths	8.95 ± 1.48 ^2^*	No deaths	No deaths	7.43 ± 2.73 ^7^	No deaths	20.0 ^1^	10.25 ± 5.36 ^8^*	10.70 ± 4.10 ^2^
	P3	2.21 ± 1.40 ^†††^	1.15 ± 0.14 ^8^	1.66 ± 0.65 ^‡‡^	3.15 ± 3.48 ^¶¶^	2.84 ± 2.80 ^5‡‡^	1.82 ± 0.91	2.57 ± 2.70 ^11^	3.13 ± 3.20 ^4‡‡^	1.41 ± 0.45 ^10^
	P3–<24 h	No deaths	7.90 ± 2.18 ^3^||	No deaths	No deaths	5.30 ^1^	6.57 ± 2.83 ^3^	12.85 ± 8.70 ^2^	7.90 ^1^	1.65 ± 0.50 ^2^
	24 h	2.79 ± 1.45 *	2.60 ± 0.19 ^5†^	2.29 ± 0.70 ^‡^	2.93 ± 0.56 ||	2.88 ± 0.83 ^4^**	2.32 ± 0.86 ^9†^	2.61 ± 0.43 ^9‡^	1.83 ± 0.21 ^3^*	1.50 ± 0.52 ^8^
	24–<48 h	7.13 ± 4.08 ^4^	5.48 ± 2.56 ^5†^	No deaths	5.30 ± 2.60 ^3^	5.53 ± 4.00 ^3^	3.80 ± 1.47 ^5§^	2.1 ^1^	No deaths	2.44 ± 0.98 ^8§^
	48 h	2.93 ± 0.76 ^6^***	No survivors	1.94 ± 0.58 ^¶^	2.19 ± 0.70 ^9^	2.6 ^1^	4.23 ± 1.73 ^4^	2.70 ± 1.42 ^8¶^	2.60 ± 0.76 ^3¶^	No survivors
	48–<72 h	No sample	No survivors	No deaths	No deaths	No deaths	4.57 ± 1.95 ^3^	4.55 ± 2.76 ^2^	2.9 ^1^	No survivors
	72 h	1.78 ± 0.49 ^5^*	No survivors	1.29 ± 0.65	3.76 ± 4.29 ^9^	1.7 ^1^	2.7 ^1^	1.30 ± 0.21 ^6^	1.55 ± 0.50 ^2^	No survivors

Measurements were taken at baseline, end of Phase 3, 24 h (lactate only), 48 h (lactate only), and 72 h (survivors), or at time of sacrifice according to humane endpoints (non-survivors) (see Section 2.6). Data represent mean ± SD. Results for P1–2, P3–<24 h, 24–<48 h, and 48–<72 h indicated by grey shading are for moribund animals sacrificed prior to experimental end (72 h), i.e., non-survivors. n values for NT, FFP, and FWB groups are n = 10, n = 12, and n = 12, respectively, or indicated by superscript number. P1, Phase 1 (60 min bolus resuscitation); P2, Phase 2 (4 h drip resuscitation); P3, Phase 3 (60 min transfusion); ALM, adenosine, lidocaine, magnesium; NT, no transfusion; FFP, fresh frozen plasma; FWB, fresh whole blood; NA, not available. No baseline measurements and reduced sample numbers for pH due to effect of anesthesia or oximetry measuring errors (indicated by NA or reduced n value). No sample for Sham NT animal sacrificed at 49 h. * *p* < 0.05 compared to baseline; ^†^ *p* < 0.05 compared to baseline and Phase 3; ^‡^ *p* < 0.05 compared to baseline, Phase 3, and 72 h; ^§^ *p* < 0.05 compared to baseline, Phase 3, and 24 h; || *p* < 0.05 compared to baseline and 48 h; ^¶^ *p* < 0.05 compared to baseline and 72 h; ** *p* < 0.05 compared to Phase 3; ^††^ *p* < 0.05 compared to Sham NT and Saline FWB; ^‡‡^ *p* < 0.05 compared to Saline NT; ^¶¶^ *p* < 0.05 compared to Sham FWB; *** *p* < 0.05 compared to ALM NT, baseline, Phase 3, and 72 h; ^†††^ *p* < 0.05 compared to baseline and Saline NT.

**Table 3:** Red blood cell parameters and platelet count for Shams, Saline controls, and ALM group without transfusion, and with FFP or FWB following laparotomy, liver resection, and hemorrhage.

Parameter [Reference Range]	Time	Sham NT	Saline NT	ALM NT	Sham FFP	Saline FFP	ALM FFP	Sham FWB	Saline FWB	ALM FWB
RBC (×10^12^/L) [5.30–10.00]	Baseline	7.90 ± 0.35 ^†^	7.52 ± 0.65 ^‡^	8.42 ± 0.46	8.72 ± 0.18	8.50 ± 1.10	879 ± 0.59	8.99 ± 0.29	8.45 ± 0.53	8.67 ± 0.52
	P1–P2	No deaths	5.38 ± 0.89 ^2^	No deaths	No deaths	6.52 ± 1.60 ^7^	No deaths	8.12 ^1^	6.08 ± 1.42 ^8^*	7.61 ± 0.32 ^2^*
	P3–<24 h	No deaths	7.35 ± 0.51 ^3^	No deaths	No deaths	5.06 ^1^	6.32 ± 0.77 ^3^	5.67 ± 0.91 ^2^	4.94 ^1^	8.01 ± 0.24 ^2^
	24–<48 h	5.57 ± 0.75 ^4^*	6.14 ± 1.53 ^5^	No deaths	6.73 ± 3.49 ^3^	8.57 ± 2.16 ^3^	6.10 ± 1.32 ^5^*	7.30 ^1^	No deaths	7.92 ± 1.33 ^8^
	48–<72 h	5.48 ^1^	No survivors	No deaths	No deaths	No deaths	7.46 ± 1.84 ^3^*	5.69 ± 0.93 ^2^	6.58 ^1^	No survivors
	72 h	5.00 ± 0.95 ^5^*	No survivors	5.63 ± 1.01 *	6.02 ± 0.83 ^9^*	5.20 ^1^	2.39 ^1^	5.77 ± 1.54 ^6^*	6.05 ± 0.79 ^2^	No survivors
HgB (g/dL) [14.0–18.0]	Baseline	15.0 ± 0.5	14.9 ± 0.6	15.4 ± 0.8	15.5 ± 1.5	14.6 ± 2.0	15.4 ± 0.6	15.6 ± 0.5	14.7 ± 1.2	15.6 ± 1.1
	P1–P2	No deaths	10.0 ± 0.3 ^2^*	No deaths	No deaths	11.3 ± 2.9 ^7^	No deaths	14.2 ^1^	10.4 ± 2.4 ^8^*	13.3 ± 0.3 ^2^
	P3–<24 h	No deaths	13.1 ± 2.6 ^3^	No deaths	No deaths	8.4 ^1^	11.2 ± 0.7 ^3^	9.5 ± 1.4 ^2^	9.3 ^1^	14.8 ± 1.2 ^2^
	24–<48 h	9.4 ± 1.1 ^4^*	11.4 ± 2.7 ^4^*	No deaths	11.4 ± 5.9 ^3^	14.5 ± 4.4 ^3^	11.0 ± 2.4 ^5^*	12.2 ^1^	No deaths	14.4 ± 2.7 ^8^
	48–<72 h	9.7 ^1^	No survivors	No deaths	No deaths	No deaths	12.5 ± 3.2 ^3^	9.5 ± 1.7 ^2^	11.7 ^1^	No survivors
	72 h	9.2 ± 1.7 ^5^*	No survivors	10.2 ± 1.7 *	10.7 ± 1.8 ^9^*	8.5 ^1^	4.1 ^1^	10.8 ± 3.5 ^6^*	10.5 ± 1.2 ^2^	No survivors
HCT (%) [35.00–52.00]	Baseline	44.87 ± 3.71 ^§^	42.64 ± 2.91 ||	48.21 ± 2.80	48.20 ± 2.44	47.20 ± 5.78	47.78 ± 2.98	49.78 ± 1.96	46.88 ± 3.90	45.98 ± 2.93
	P1–P2	No deaths	29.27 ± 5.06 ^2^	No deaths	No deaths	34.28 ± 7.89 ^7^	No deaths	45.63 ^1^	32.71 ± 7.25 ^8^*	38.35 ± 0.07 ^2^*
	P3–<24 h	No deaths	40.90 ± 2.96 ^3^	No deaths	No deaths	25.32 ^1^	33.51 ± 4.83 ^3^*	30.34 ± 6.57 ^2^	26.26 ^1^	40.72 ± 1.24 ^2^*
	24–<48 h	29.31 ± 5.26 ^4^	34.75 ± 8.85 ^4^	No deaths	36.40 ± 19.88 ^3^	46.83 ± 11.45 ^3^	32.45 ± 6.88 ^5^*	39.46 ^1^	No deaths	41.93 ± 8.00 ^8^
	48–<72 h	29.43 ^1^	No survivors	No deaths	No deaths	No deaths	40.23 ± 11.41 ^3^	31.01 ± 6.18 ^2^	37.15 ^1^	No survivors
	72 h	28.23 ± 5.24 ^5^*	No survivors	32.03 ± 5.29 *	33.05 ± 4.03 ^9^*	28.91 ^1^	13.44 ^1^	32.42 ± 9.31 ^6^*	32.75 ± 5.28 ^2^	No survivors
MCHC (g/dL) [31.0–40.0]	Baseline	33.5 ± 2.2	35.1 ± 2.9^¶^	31.9 ± 1.0	32.2 ± 3.9	30.9 ± 1.5	32.2 ± 1.4	31.4 ± 0.9	31.4 ± 0.9	34.0 ± 2.0 ^§^
	P1–P2	No deaths	34.6 ± 5.2 ^2^	No deaths	No deaths	32.8 ± 1.6 ^7^*	No deaths	31.2 ^1^	31.5 ± 1.8 ^8^	34.8 ± 0.6 ^2^
	P3–<24 h	No deaths	32.0 ± 5.3 ^3^	No deaths	No deaths	33.2 ^1^	33.6 ± 3.3 ^3^	31.7 ± 2.1 ^2^	35.4 ^1^	36.1 ± 1.8 ^2^
	24–<48 h	32.5 ± 2.5 ^4^	32.9 ± 1.9 ^4^	No deaths	31.9 ± 2.0 ^3^	30.7 ± 1.7 ^3^	33.7 ± 0.9 ^5^	30.8 ^1^	No deaths	34.3 ± 2.1 ^8^
	48–<72 h	33.1 ^1^	No survivors	No deaths	No deaths	No deaths	31.2 ± 1.2 ^3^	30.7 ± 0.8 ^2^	31.4 ^1^	No survivors
	72 h	32.7 ± 2.9 ^5^	No survivors	32.1 ± 1.2 *	32.2 ± 2.5	29.3 ^1^	30.6 ^1^	33.4 ± 5.7 ^6^	32.0 ± 1.5 ^2^	No survivors
PLT (×10^9^/L) [100–500]	Baseline	279 ± 115	300 ± 111	135 ± 71	123 ± 113 **	163 ± 149	202 ± 138	242 ± 122	154 ± 133	175 ± 129
	P1–P2	No deaths	257 ± 11 ^2^	No deaths	No deaths	256 ± 121 ^7^*	No deaths	7 ^1^	248 ± 179 ^8^	227 ± 168 ^2^
	P3–<24 h	No deaths	52 ± 25 ^3^	No deaths	No deaths	162 ^1^	207 ± 122 ^3^	149 ± 119 ^2^	241 ^1^	203 ± 90 ^2^
	24–<48 h	54 ± 35 ^4^	18 ± 19 ^4^*	No deaths	54 ± 75 ^3^	36 ± 51 ^3^	169 ± 138 ^5^	0 ^1^	No deaths	114 ± 98 ^8^
	48–<72 h	20 ^1^	No survivors	No deaths	No deaths	No deaths	15 ± 4 ^3^	11 ± 16 ^2^	17 ^1^	No survivors
	72 h	131 ± 61 ^5^*	No survivors	125 ± 100	79 ± 71 ^9^	120 ^1^	94 ^1^	114 ± 66 ^6^	145 ± 32 ^2^	No survivors
PCT (%)	Baseline	0.21 ± 0.08	0.21 ± 0.08	0.10 ± 0.05	0.09 ± 0.08 **	0.11 ± 0.10	0.14 ± 0.10	0.18 ± 0.06	0.11 ± 0.09	0.12 ± 0.07
	P1–P2	No deaths	0.17 ± 0.02 ^2^	No deaths	No deaths	0.15 ± 0.07 ^7^	No deaths	0.00 ^1^	0.14 ± 0.10 ^8^	0.14 ± 0.10 ^2^
	P3–<24 h	No deaths	0.04 ± 0.02 ^3^	No deaths	No deaths	0.09 ^1^	0.13 ± 0.08 ^3^*	0.09 ± 0.07 ^2^	0.14 ^1^	0.13 ± 0.06 ^2^
	24–<48 h	0.04 ± 0.03 ^4^	0.01 ± 0.02 ^4^*	No deaths	0.03 ± 0.04 ^3^	0.06± 0.06 ^3^	0.11 ± 0.10 ^5^	Not calculable	No deaths	0.07 ± 0.07 ^8^
	48–<72 h	0.01 ^1^	No survivors	No deaths	No deaths	No deaths	0.01 ± 0.00 ^3^	0.01 ^1^	0.01 ^1^	No survivors
	72 h	0.10 ± 0.05 ^5^	No survivors	0.10 ± 0.07	0.06 ± 0.06 ^9^	0.07 ^1^	0.06 ^1^	0.08 ± 0.05 ^6^	0.10 ± 0.01 ^2^	No survivors

Measurements were taken at baseline and 72 h (survivors), or at time of sacrifice according to humane endpoints (non-survivors) (see Section 2.6). Data represent mean ± SD. Results for P1–P2, P3–<24 h, 24–<48 h, and 48–<72 h indicated by grey shading are for moribund animals sacrificed prior to experimental end (72 h), i.e., non-survivors. n values for NT, FFP, and FWB groups are n = 10, n = 12, and n = 12, respectively, or indicated by superscript number. P1, Phase 1 (60 min bolus resuscitation); P2, Phase 2 (4 h drip resuscitation); P3, Phase 3 (60 min transfusion); ALM, adenosine, lidocaine, magnesium; NT, no transfusion; FFP, fresh frozen plasma; FWB, fresh whole blood; RBC, red blood cell; HgB, hemoglobin; HCT, hematocrit; MCHC, mean corpuscular hemoglobin concentration (average [HgB]/RBC); PLT, platelet; PCT, plateletcrit (total platelet mass); * *p* < 0.05 compared to baseline; ^†^ *p* < 0.05 compared to Sham FFP and Sham FWB; ^‡^ *p* < 0.05 compared to ALM NT, Saline FFP, and Saline FWB; ^§^ *p* < 0.05 compared to Sham FWB; || *p* < 0.05 compared to ALM NT; ^¶^ *p* < 0.05 compared to ALM NT, Sham FFP, and Sham FWB; ** *p* < 0.05 compared to Sham NT.

**Table 4:** White blood cell differential in Shams, Saline controls, and ALM group without transfusion, and with FFP or FWB following laparotomy, liver resection, and hemorrhage.

Parameter [Reference Range]	Time	Sham NT	Saline NT	ALM NT	Sham FFP	Saline FFP	ALM FFP	Sham FWB	Saline FWB	ALM FWB
WBC (×10^9^/L) [2.10–19.50]	Baseline	8.02 ± 3.10	8.92 ± 2.05	12.19 ± 2.80	10.24 ± 3.07	9.85 ± 3.62	11.35 ± 4.02	11.79 ± 3.92	11.31 ± 3.91	10.56 ± 3.91
	P1–P2	No deaths	4.04 ± 3.48 ^2^	No deaths	No deaths	7.12 ± 5.33 ^7^	No deaths	8.42 ^1^	3.87 ± 3.14 ^8^*	5.76 ± 4.60 ^2^
	P3–<24 h	No deaths	2.31 ± 1.31 ^3^	No deaths	No deaths	8.07 ^1^	7.09 ± 3.27 ^3^	9.46 ± 2.92 ^2^	1.79 ^1^	8.69 ± 1.67 ^2^
	24–<48 h	4.48 ± 6.76 ^4^	0.76 ± 0.39 ^5^*	No deaths	1.83 ± 1.13 ^3^*	2.46 ± 2.33 ^3^*	2.85 ± 1.64 ^5^*	1.34 ^1^	No deaths	1.89 ± 1.80 ^8^*
	48–<72 h	0.56 ^1^	No survivors	No deaths	No deaths	No deaths	1.38 ± 1.55 ^3^*	0.73 ± 0.12 ^2^	2.63 ^1^	No survivors
	72 h	6.03 ± 3.79 ^5^	No survivors	10.68 ± 4.08	11.38 ± 5.35 ^9^	4.84 ^1^	1.29 ^1^	6.10 ± 4.13 ^6^*	7.28 ± 5.12 ^2^	No survivors
LYM (×10^9^/L) [2.00–14.10]	Baseline	5.76 ± 1.98	6.65 ± 1.17	8.62 ± 2.12	7.59 ± 2.42	7.07 ± 2.23	7.62 ± 2.46	8.81 ± 3.37	7.80 ± 2.83	6.39 ± 2.40
	P1–P2	No deaths	1.65 ± 1.00 ^2^	No deaths	No deaths	2.82 ± 1.80 ^7^*	No deaths	8.42 ^1^	1.94 ± 1.75 ^8^*	1.42 ± 0.66 ^2^*
	P3–<24 h	No deaths	0.75 ± 0.36 ^3^*	No deaths	No deaths	4.11 ^1^	2.65 ± 2.31 ^3^	4.95 ± 2.58 ^2^	0.71 ^1^	2.75 ± 1.29 ^2^
	24–<48 h	0.92 ± 0.72 ^4^*	0.52 ± 0.31 ^4^*	No deaths	1.02 ± 0.45 ^3^*	0.29 ± 0.21 ^3^*	1.15 ± 0.53 ^5^*	0.67 ^1^	No deaths	0.89 ± 0.48 ^8^*
	48–<72 h	0.42 ^1^	No survivors	No deaths	No deaths	No deaths	0.53 ± 0.58 ^3^*	0.51 ± 0.25	1.05 ^1^	No survivors
	72 h	1.92 ± 0.79 ^5^*	No survivors	4.66 ± 2.78 *	5.12 ± 2.66 ^9^*	1.75 ^1^	0.97 ^1^	1.71 ± 1.78 ^6^*	3.45 ± 2.89 ^2^*	No survivors
MON (×10^9^/L) [0.00–0.98]	Baseline	0.55 ± 0.44	0.39 ± 0.31	0.59 ± 0.64	0.57 ± 0.61	0.69 ± 0.50	0.84 ± 0.59	0.67 ± 0.68	0.81 ± 0.53 ^1^	0.41 ± 0.62
	P1–P2	No deaths	0.32 ± 0.33 ^2^	No deaths	No deaths	0.35 ± 0.32 ^7^	No deaths	0.89 ^1^	0.26 ± 0.25 ^8^	0.40 ± 0.31 ^2^
	P3–<24 h	No deaths	0.15 ± 0.09 ^3^	No deaths	No deaths	0.90 ^1^	0.53 ± 0.31 ^3^*	0.54 ± 0.04 ^2^	0.21 ^1^	0.67 ± 0.02 ^2^
	24–<48 h	0.38 ± 0.69 ^4^	0.04 ± 0.03 ^4^	No deaths	0.12 ± 0.11 ^3^	0.24 ± 0.29 ^3^	0.21 ± 0.27 ^5^	0.13 ^1^	No deaths	0.10 ± 0.11 ^8^*
	48–<72 h	0.03 ^1^	No survivors	No deaths	No deaths	No deaths	0.07 ± 0.07 ^3^	0.03 ± 0.04 ^2^	0.24 ^1^	No survivors
	72 h	0.34 ± 0.27 ^5^	No survivors	0.95 ± 0.73	0.77 ± 0.49 ^9^	0.15 ^1^	0.01 ^1^	0.37 ± 0.27 ^6^	0.19 ± 0.07 ^2^	No survivors
NEU (×10^9^/L) [0.10–5.40]	Baseline	1.72 ± 0.99	1.89 ± 1.09	2.98 ± 1.22	2.08 ± 1.03	2.10 ± 1.22	2.89 ± 1.39	2.31 ± 0.85	2.68 ± 1.01	2.53 ± 1.38
	P1–P2	No deaths	2.07 ± 2.14 ^2^	No deaths	No deaths	3.95 ± 3.71 ^7^	No deaths	2.72 ^1^	2.04 ± 1.70 ^8^	3.94 ± 3.63 ^2^
	P3–<24 h	No deaths	1.42 ± 1.50 ^3^	No deaths	No deaths	3.06 ^1^	3.91 ± 1.60 ^3^	3.98 ± 0.37 ^2^	0.87 ^1^	5.27 ± 0.40 ^2^
	24–<48 h	3.18 ± 5.65 ^4^	0.33 ± 0.18 ^4^*	No deaths	0.69 ± 0.57 ^3^	1.93 ± 1.84 ^3^	1.50 ± 1.30 ^5^	0.54 ^1^	No deaths	0.91 ± 1.58 ^8^*
	48–<72 h	0.11 ^1^	No survivors	No deaths	No deaths	No deaths	0.77 ± 0.93 ^3^	0.20 ± 0.11 ^2^	1.34 ^1^	No survivors
	72 h	3.76 ± 2.96 ^5^	No survivors	5.07 ± 0.96 *	5.50 ± 2.62 ^9^*	2.94 ^1^	0.31 ^1^	4.03 ± 2.13 ^6^	3.65 ± 2.16 ^2^	No survivors
LYM (%)	Baseline	72.5 ± 7.7	75.5 ± 6.8	71.1 ± 8.1	75.0 ± 9.3	73.6 ± 8.6	68.6 ± 7.2	74.0 ± 8.7	69.1 ± 7.5	70.1 ± 7.1
	P1–P2	No deaths	47.9 ± 16.2 ^2^	No deaths	No deaths	47.7 ± 20.9 ^7^*	No deaths	57.1 ^1^	45.5 ± 19.3 ^8^*	29.5 ± 12.0 ^2^
	P3–<24 h	No deaths	43.7 ± 30.5 ^3^	No deaths	No deaths	50.9 ^1^	34.0 ± 17.0 ^3^	50.5 ± 11.7 ^2^	39.5 ^1^	30.9 ± 8.8 ^2^
	24–<48 h	46.3 ± 30.4 ^4^	58.2 ± 23.7 ^4^	No deaths	60.8 ± 15.0 ^3^	14.2 ± 5.9 ^3^*	49.8 ± 27.0 ^5^	49.6 ^1^	No deaths	65.1 ± 24.7 ^8^
	48–<72 h	74.5 ^1^	No survivors	No deaths	No deaths	No deaths	46.6 ± 29.1 ^3^	68.1 ± 23.3 ^2^	39.8 ^1^	No survivors
	72 h	34.9 ± 10.4 ^5^*	No survivors	40.0 ± 13.8 *	44.2 ± 5.9 ^9^*	36.2 ^1^	75.3 ^1^	23.7 ± 11.3 ^6†^	44.4 ± 8.6 ^2^	No survivors
MON (%)	Baseline	3.9 ± 4.0	4.3 ± 3.5	5.0 ± 4.8	5.2 ± 5.1	6.5 ± 3.7	7.0 ± 4.0	5.9 ± 5.0	6.7 ± 3.7	6.7 ± 3.1
	P1–P2	No deaths	7.0 ± 2.1 ^2^	No deaths	No deaths	4.9 ± 2.5 ^7^	No deaths	10.5 ^1^	6.4 ± 3.9 ^8^	7.1 ± 0.3 ^2^
	P3–<24 h	No deaths	6.3 ± 0.4 ^3^	No deaths	No deaths	11.2 ^1^	7.0 ± 1.4 ^3^	6.0 ± 2.3 ^2^	11.7 ^1^	7.8 ± 1.7 ^2^
	24–<48 h	5.5 ± 3.9 ^4^	3.9 ± 2.6 ^4^	No deaths	6.0 ± 2.0 ^3^	8.8 ± 3.8 ^3^	5.9 ± 4.6 ^5^	9.8 ^1^	No deaths	4.5 ± 2.0 ^8^
	48–<72 h	6.1 ^1^	No survivors	No deaths	No deaths	No deaths	6.0 ± 6.6 ^3^	3.9 ± 4.6 ^2^	9.2 ^1^	No survivors
	72 h	5.5 ± 3.4 ^5^	No survivors	8.4 ± 3.9 *	6.6 ± 1.9 ^9^	3.1 ^1^	0.6 ^1^	5.9 ± 1.4 ^6^	3.1 ± 1.2 ^2^	No survivors
NEU (%)	Baseline	20.5 ± 5.0	20.2 ± 6.7	23.9 ± 7.6	19.9 ± 7.2	19.9 ± 6.8	24.4 ± 5.2	20.1 ± 5.5	24.2 ± 6.5	23.3 ± 6.0
	P1–P2	No deaths	45.2 ± 14.1 ^2^	No deaths	No deaths	47.4 ± 20.3 ^7^*	No deaths	32.3 ^1^	48.1 ± 17.0 ^8^*	63.5 ± 12.3 ^2^
	P3–<24 h	No deaths	50.1 ± 30.1 ^3^	No deaths	No deaths	37.9 ^1^	58.9 ± 18.0 ^3^	43.5 ± 9.5 ^2^	48.8 ^1^	61.4 ± 7.1 ^2^*
	24–<48 h	48.2 ± 29.4 ^4^	37.9 ± 21.4 ^4^	No deaths	33.2 ± 13.4 ^3^	76.9 ± 8.3 ^3^*	44.3 ± 24.1 ^5^	40.5 ^1^	No deaths	30.3 ± 24.2 ^8^
	48–<72 h	19.4 ^1^	No survivors	No deaths	No deaths	No deaths	47.4 ± 23.5 ^3^	28.2 ± 18.7 ^2^	51.0 ^1^	No survivors
	72 h	59.7 ± 11.8 ^5^*	No survivors	51.6 ± 14.3 *	49.2 ± 7.2 ^9^*	60.7 ^1^	24.2 ^1^	70.5 ± 11.3 ^6†^	52.6 ± 7.4 ^2^	No survivors
NEU:LYM	Baseline	0.29 ± 0.10	0.28 ± 0.13	0.35 ± 0.14	0.28 ± 0.13	0.28 ± 0.13	0.37 ± 0.11	0.28 ± 0.11	0.36 ± 0.13	0.71 ± 1.31
	P1–P2	No deaths	0.05 ± 0.06 ^2^*	No deaths	No deaths	0.12 ± 0.13 ^7^	No deaths	0.05 ^1^	0.53 ± 0.04 ^8^*	0.17 ± 0.19 ^2^
	P3–<24 h	No deaths	3.20 ± 4.54 ^3^	No deaths	No deaths	0.74 ^1^	2.16 ± 1.28 ^3^	0.91 ± 0.40 ^2^	1.23 ^1^	2.11 ± 0.84 ^2^
	24–<48 h	2.59 ± 3.47 ^4^	1.00 ± 1.13 ^4^	No deaths	0.69 ± 0.33 ^3^	6.04 ± 2.49 ^3‡^	1.43 ± 1.32 ^5^	0.81 ^1^	No deaths	1.02 ± 1.82 ^8^
	48–<72 h	0.26 ^1^	No survivors	No deaths	No deaths	No deaths	1.53 ± 1.26 ^3^	0.50 ± 0.46 ^2^	1.28 ^1^	No survivors
	72 h	1.90 ± 0.84 ^5^*	No survivors	1.84 ± 1.95 *	1.15 ± 0.30 ^9^*	1.68 ^1^	0.32 ^1^	4.54 ± 4.36 ^6^	1.23 ± 0.40 ^2^	No survivors
MON:NEU	Baseline	0.33 ± 0.18	0.23 ± 0.20	0.24 ± 0.22	0.27 ± 0.27	0.36 ± 0.20	0.29 ± 0.15	0.29 ± 0.26	0.29 ± 0.14	0.31 ± 0.14
	P1–P2	No deaths	0.16 ± 0.01 ^2^	No deaths	No deaths	0.13 ± 0.13 ^7^*	No deaths	0.33 ^1^	0.14 ± 0.08 ^8^	0.11 ± 0.03 ^2^
	P3–<24 h	No deaths	0.16 ± 0.08 ^3^	No deaths	No deaths	0.29 ^1^	0.13 ± 0.07 ^3^*	0.14 ± 0.02 ^2^	0.24 ^1^	0.13 ± 0.01 ^2^
	24–<48 h	0.15 ± 0.10 ^4^	0.10 ± 0.07 ^4^	No deaths	0.19 ± 0.06 ^3^	0.12 ± 0.06 ^3^	0.15 ± 0.10 ^5^	0.24 ^1^	No deaths	0.23 ± 0.18 ^8^
	48–<72 h	0.27 ^1^	No survivors	No deaths	No deaths	No deaths	0.10 ± 0.11 ^3^	0.09 ± 0.13 ^2^	0.18 ^1^	No survivors
	72 h	0.10 ± 0.07 ^5^*	No survivors	0.18 ± 0.11	0.14 ± 0.06 ^9^	0.05 ^1^	0.03 ^1^	1.16 ± 2.63 ^6^	0.06 ± 0.07 ^2^	No survivors

Measurements were taken at baseline and 72 h (survivors), or at time of sacrifice according to humane endpoints (non-survivors) (see Section 2.6). Data represent mean ± SD. Results for P1–P2, P3–<24 h, 24–<48 h, and 48–<72 h indicated by grey shading are for moribund animals sacrificed prior to experimental end (72 h), i.e., non-survivors. n values for NT, FFP, and FWB groups are n = 10, n = 12, and n = 12, respectively, or indicated by superscript number. P1, Phase 1 (60 min bolus resuscitation); P2, Phase 2 (4 h drip resuscitation); P3, Phase 3 (60 min transfusion); ALM, adenosine, lidocaine, magnesium; NT, no transfusion; FFP, fresh frozen plasma; FWB, fresh whole blood; WBC, white blood cell; LYM, lymphocyte; MON, monocyte; NEU, neutrophil; NEU:LYM, neutrophil:lymphocyte ratio calculated from total neutrophils divided by total lymphocytes; MON:NEU, monocyte:neutrophil ratio calculated from total monocytes divided by total neutrophils. * *p* < 0.05 compared to baseline; ^†^ *p* < 0.05 compared to baseline and Sham FFP; ^‡^ *p* < 0.05 compared to Saline NT and ALM FFP.

**Table 5:** Coagulation parameters measured in plasma for Shams, Saline controls, and ALM group without transfusion, and with FFP or FWB following laparotomy, liver resection, and hemorrhage.

Parameter	Time	Sham NT	Saline NT	ALM NT	Sham FFP	Saline FFP	ALM FFP	Sham FWB	Saline FWB	ALM FWB
PT (s)	Baseline	16.5 ± 2.0 ^11^	16.5 ± 2.0 ^11^	16.5 ± 2.0 ^11^	16.5 ± 2.0 ^11^	16.5 ± 2.0 ^11^	16.5 ± 2.0 ^11^	16.5 ± 2.0 ^11^	16.5 ± 2.0 ^11^	16.5 ± 2.0 ^11^
	P1–P2	No deaths	25.3 ^1^	No deaths	No deaths	46.0 ± 45.1 ^7^	No deaths	180.1 ^1^	62.4 ± 65.0 ^8^*	108.3 ± 53.6 ^2^
	P3–<24 h	No deaths	21.0 ^1^	No deaths	No deaths	24.0 ^1^	27.5 ± 4.0 ^3^	102.6 ± 109.6 ^2^	30.5 ^1^	19.5 ± 2.5 ^2^
	24 h	22.1 ± 8.5 ^9†^	20.0 ± 1.7 ^4^	19.2 ± 2.2 ^5†^	18.9 ± 3.3 ^11†^	20.5 ± 1.8 ^4^	17.6 ± 2.3 ^8^	17.8 ± 4.2 ^9^	21.0 ± 3.0 ^3^	19.0 ± 1.4 ^6^
	24–<48 h	19.5 ± 4.5 ^4^	24.5 ± 12.0 ^5^	No deaths	25.5 ± 9.9 ^3^	21.4 ± 4.6 ^3^	18.5 ± 2.7 ^5^	15.3 ^1^	No deaths	18.1 ± 1.8 ^8^
	48–<72 h	14.9 ^1^	No survivors	No deaths	No deaths	No deaths	27.1 ± 8.3 ^3^	15.4 ± 0.6 ^2^	16.3 ^1^	No survivors
	72 h	14.9 ± 1.1 ^4^	No survivors	18.7 ± 4.6 ^7^	15.1 ± 0.9 ^9^	15.4 ^1^	15.8 ^1^	15.7 ± 0.8 ^6^	15.6 ± 1.3 ^2^	No survivors
aPTT (s)	Baseline	76.4 ± 32.1 ^8^	76.4 ± 32.1 ^8^	76.4 ± 32.1 ^8^	76.4 ± 32.1 ^8^	76.4 ± 32.1 ^8^	76.4 ± 32.1 ^8^	76.4 ± 32.1 ^8^	76.4 ± 32.1 ^8^	76.4 ± 32.1 ^8^
	P1–P2	No deaths	200.1 ^1^	No deaths	No deaths	200.1 ± 0 ^4^*	No deaths	NA	182.7 ± 39.0 ^5^*	200.1 ± 0 ^2^*
	P3–<24 h	No deaths	94.1 ^1^	No deaths	No deaths	200.1 ^1^	63.3 ^1^	200.1 ^1^	200.1 ^1^	45.6 ± 3.6 ^2^
	24 h	200.1 ± 0 ^7‡^	200.1 ± 0 ^4^*	200.1 ± 0 ^4‡^	195.8 ± 11.4 ^7^*	200.1 ± 0 ^2^	186.0 ± 28.2 ^4^	175.7 ± 54.5 ^6^	200.1 ± 0 ^3^*	103.5 ± 51.7 ^6^
	24–<48 h	141.3 ± 77.8 ^4^	199.7 ± 0.8 ^3^	No deaths	184.8 ^1^	200.1 ^1^	178.6 ± 30.5 ^2^	NA	No deaths	90.7 ± 76.0 ^7^
	48–<72 h	NA	No survivors	No deaths	No deaths	No deaths	NA	148.8 ± 72.5 ^2^*	117.0 ^1^	No survivors
	72 h	88.0 ± 67.4 ^4^	No survivors	57.4 ± 23.5 ^4^	126.9 ± 60.9 ^6^	200.1 ^1^	176.5 ^1^	98.3 ± 59.0 ^5^	NA	No survivors
Fibrinogen (g/dL)	Baseline	2.43 ± 0.31 ^11^	2.43 ± 0.31 ^11^	2.43 ± 0.31 ^11^	2.43 ± 0.31 ^11^	2.43 ± 0.31 ^11^	2.43 ± 0.31 ^11^	2.43 ± 0.31 ^11^	2.43 ± 0.31 ^11^	2.43 ± 0.31 ^11^
	P1–P2	No deaths	1.60 ± 0.03 ^2^*	No deaths	No deaths	1.17 ± 0.47 ^6^*	No deaths	NA	1.30 ± 0.37 ^6^*	1.05 ± 1.09 ^2^
	P3–<24 h	No deaths	3.17 ± 1.99 ^2^	No deaths	No deaths	1.01 ^1^	2.11 ± 1.62 ^3^	0.70 ± 0.54 ^2^	1.06 ^1^	2.65 ± 1.65 ^2^
	24 h	5.14 ± 0.78 ^8^*	4.63 ± 0.89 ^4^*	4.83 ± 1.39 ^9^*	5.47 ± 0.81 ^11^*	4.64 ± 0.54 ^4^*	4.99 ± 0.65 ^8^*	5.11 ± 0.65 ^9‡^	5.19 ± 0.39 ^3^*	5.32 ± 0.44 ^6^*
	24–<48 h	4.07 ± 1.81 ^4^*	4.25 ± 2.41 ^5^*	No deaths	4.56 ± 1.51 ^3^*	4.44 ± 1.51 ^3^*	4.67 ± 1.53 ^5^*	6.65 ^1^	No deaths	5.23 ± 1.35 ^8^*
	48–<72 h	6.68 ^1^	No survivors	No deaths	No deaths	No deaths	4.20 ± 1.61 ^2^	5.64 ± 0.60 ^2^*	4.40 ^1^	No survivors
	72 h	6.77 ± 0.61 ^5^*	No survivors	6.03 ± 1.03 ^8^*	5.94 ± 1.05 ^9^*	6.24 ^1^	2.91 ^1^	6.54 ± 0.77 ^6^*	6.55 ± 0.30 ^2^*	No survivors
TAFI (%)	Baseline	62 ± 35 ^11^	62 ± 35 ^11^	62 ± 35 ^11^	62 ± 35 ^11^	62 ± 35 ^11^	62 ± 35 ^11^	62 ± 35 ^11^	62 ± 35 ^11^	62 ± 35 ^11^
	P1–P2	No deaths	68 ^1^	No deaths	No deaths	59 ± 7 ^7^	No deaths	66 ^1^	69 ± 9 ^8^	36 ± 33 ^2^
	P3–<24 h	No deaths	39 ^1^	No deaths	No deaths	NA	49 ± 18 ^3^	59 ± 6 ^2^	62 ^1^	25 ± 17 ^2^
	24 h	37 ± 27 ^5^	54 ± 25 ^4^	112 ± 17 ^8§^	60 ± 28 ^11^	43 ± 14 ^4^	58 ± 14 ^7^	82 ± 20 ^9†^	90 ± 7 ^3^	19 ± 9 ^6¶^
	24–<48 h	26 ± 9 ^3^	7 ^1^	No deaths	45 ± 22 ^3^	39 ± 13 ^2^	36 ± 15 ^4^	63 ^1^	No deaths	21 ± 27 ^6^
	48–<72 h	NA	No survivors	No deaths	No deaths	No deaths	33 ± 21 ^3^	79 ± 22 ^2^	116 ^1^	No survivors
	72 h	79 ± 12 ^3^	No survivors	119 ^1^	93 ± 37 ^9^	133 ^1^	86 ^1^	125 ± 9 ^5^**	135 ^1^	No survivors
Protein C (%)	Baseline	3 ± 0 ^10^	3 ± 0 ^10^	3 ± 0 ^10^	3 ± 0 ^10^	3 ± 0 ^10^	3 ± 0 ^10^	3 ± 0 ^10^	3 ± 0 ^10^	3 ± 0 ^10^
	P1–P2	No deaths	3 ^1^	No deaths	No deaths	12.9 ± 11.8 ^7^*	No deaths	38 ^1^	10.2 ± 10.5 ^6^	9 ^1^
	P3–<24 h	No deaths	2 ± 2.8 ^2^	No deaths	No deaths	3 ^1^	10.3 ± 11.8 ^3^	9 ± 12.7 ^2^	2 ^1^	0.5 ± 0.7 ^2^
	24 h	3.1 ± 0.4 ^8^	3 ± 0 ^4^	2.6 ± 2.1 ^10^	3.1 ± 1.9 ^11^	1.8 ± 0.5 ^4^	1.4 ± 1.3 ^8^	0.2 ± 0.4 ^9^**	0 ± 0 ^3^**	0 ± 0 ^6‡^
	24–<48 h	3 ± 0 ^4^	3 ± 0 ^5^	No deaths	4.5 ± 2.1 ^2^	5 ± 0 ^2^	2.6 ± 1.5 ^5^	3 ^1^	No deaths	0.3 ± 0.5 ^6¶^
	48–<72 h	3 ^1^	No survivors	No deaths	No deaths	No deaths	2 ± 1.7 ^3^	0.5 ± 0.7 ^2^*	1 ^1^	No survivors
	72 h	5.5 ± 3.1 ^4‡‡^	No survivors	2.1 ± 2.4 ^9^	3 ± 0 ^9^	3 ^1^	7 ^1^	1.5 ± 1.4 ^6§§^	3.5 ± 0.7 ^2^	No survivors
Antiplasmin (%)	Baseline	156 ± 51 ^11^	156 ± 51 ^11^	156 ± 51 ^11^	156 ± 51 ^11^	156 ± 51 ^11^	156 ± 51 ^11^	156 ± 51 ^11^	156 ± 51 ^11^	156 ± 51 ^11^
	P1–P2	No deaths	115 ^1^	No deaths	No deaths	107 ± 17 ^7^*	No deaths	110 ^1^	95 ± 22 ^7^*	131 ^1^
	P3–<24 h	No deaths	112 ^1^	No deaths	No deaths	115 ^1^	83 ± 5 ^3^||	95 ± 16 ^2^*	101 ^1^	159 ± 50 ^2^
	24 h	101 ± 10 ^6†††^	119 ± 2 ^4^	152 ± 46 ^3^	179 ± 35 ^11¶¶^	129 ± 49 ^3^	101 ± 4 ^8^***	146 ± 42 ^9^	125 ± 5 ^3^	126 ± 3 ^6^
	24–<48 h	103 ± 7 ^4‡‡‡^	127 ± 4 ^3^	No deaths	151 ± 110 ^3^	85 ± 27 ^2‡‡‡^	101 ± 9 ^5‡‡‡^	122 ^1^	No deaths	127 ± 3 ^6^
	48–<72 h	NA	No survivors	No deaths	No deaths	No deaths	116 ± 39 ^3^	139 ± 93 ^2^	121 ^1^	No survivors
	72 h	113 ± 3 ^4^	No survivors	230 ± 8 ^3§§§^	216 ± 16 ^9§§§^	227 ^1^	52 ^1^	202 ± 21 ^6^	177 ± 16 ^2^	No survivors

Measurements were taken at 24 h and 72 h, or time of sacrifice, according to humane endpoints (non-survivors) (refer to Section 2.6). Data represent mean ± SD. n value indicated by superscript number. Samples were run on the STA Compact (Diagnostica Stago) in the following order: PT, fibrinogen, TAFI, Antiplasmin, Protein C, aPTT, with some results not available due to insufficient sample (indicated by NA or reduced n value). Results for P1–P2, P3–<24 h, 24–<48 h, and 48–<72 h indicated by grey shading are for moribund animals sacrificed prior to experimental end (72 h), i.e., non-survivors. Baseline values taken from healthy male Sprague-Dawley rats. P1, Phase 1 (60 min bolus resuscitation); P2, Phase 2 (4 h drip resuscitation); P3, Phase 3 (60 min transfusion); ALM, adenosine, lidocaine, magnesium; NT, no transfusion; FFP, fresh frozen plasma; FWB, fresh whole blood; NA, not available; PT, prothrombin time; aPTT, activated partial thromboplastin time; TAFI, tissue activatable fibrinolysis inhibitor. Maximum recorded time for PT was 180.1 s, and for aPTT was 200.1 s. * *p* < 0.05 compared to baseline; ^†^ *p* < 0.05 compared to 72 h; ^‡^ *p* < 0.05 compared to baseline and 72 h; ^§^ *p* < 0.05 compared to baseline, Sham NT, ALM FFP, and ALM FWB; ^¶^ *p* < 0.05 compared to ALM NT, ALM FFP, Sham FWB, and Saline FWB; ** *p* < 0.05 compared to baseline, Sham NT, and Sham FFP; ^‡‡^ *p* < 0.05 compared to baseline, ALM NT, Sham FFP, and Sham FWB; ^§§^ *p* < 0.05 compared to baseline, Sham NT, Sham FFP, and Saline FWB; || *p* < 0.05 compared to baseline and ALM FWB; ^¶¶^ *p* < 0.05 compared to Sham NT and ALM FFP; *** *p* < 0.05 compared to Sham FFP, and ALM FWB; ^†††^ *p* < 0.05 compared to Sham FFP; ^‡‡‡^ *p* < 0.05 compared to baseline, Saline NT, and ALM FWB; ^§§§^ *p* < 0.05 compared to baseline and Sham NT.

**Table 6:** ROTEM parameters for Shams, Saline controls, and ALM group without transfusion, and with FFP or FWB following hemorrhage.

Test	Parameter	Time	Sham NT	Saline NT	ALM NT	Sham FFP	Saline FFP	ALM FFP	Sham FWB	Saline FWB	ALM FWB
EXTEM	CT (s)	Baseline	42 ± 3	42 ± 3	42 ± 3	42 ± 3	42 ± 3	42 ± 3	42 ± 3	42 ± 3	42 ± 3
		P1–P2	No deaths	48 ± 7 ^2^	No deaths	No deaths	94 ± 108 ^7^*	No deaths	DNC	202 ± 367 ^8^*	253 ± 255 ^2^*
		P3	53 ± 27	50 ± 6 ^8^	51 ± 9	45 ± 6	44 ± 4 ^5^	54 ± 6 *	45 ± 5 ^9^	39 ± 3 ^3^	65 ± 9 ^10‡^
		P3–<24 h	No deaths	135 ± 77 ^3^	No deaths	No deaths	43 ^1^	50 ± 10 ^3^	59 ± 4 ^2^	41 ^1^	77 ± 25 ^2^
		24–<48 h	49 ± 9 ^4^	55 ± 7 ^5^ n = 1 DNC	No deaths	89 ± 34 ^3^	55 ± 1 ^3^	56 ± 8 ^5^	42 ^1^	No deaths	65 ± 7 ^8^
		48 h	41 ± 3 ^5^	No survivors	47 ± 5	43 ± 3 ^9^	48 ^1^	46 ^1^	51 ± 12 ^8^	51 ± 11 ^3^	No survivors
		48–<72 h	36 ^1^	No survivors	No deaths	No deaths	No deaths	187 ± 199 ^3^	48 ± 9 ^2^	62 ^1^	No survivors
		72 h	47 ± 8 ^5^	No survivors	43 ± 4	42 ± 3 ^9^	48 ^1^	45 ^1^	46 ± 4 ^6^	47 ± 11 ^2^	No survivors
	MCF (mm)	Baseline	77 ± 1	77 ± 1	77 ± 1	77 ± 1	77 ± 1	77 ± 1	77 ± 1	77 ± 1	77 ± 1
		P1–P2	No deaths	69 ± 1 ^2^*	No deaths	No deaths	59 ± 22 ^7^*	No deaths	DNC	55 ± 26 ^8^*	36 ± 40 ^2^*
		P3	66 ± 18	70 ± 4 ^8^	72 ± 3	73 ± 3 ^12^	69 ± 2 ^5^	72 ± 3 ^12^	73 ± 1 ^9^	73 ± 3 ^3^	71 ± 3 ^10^
		P3–<24 h	No deaths	30 ± 33 ^3^	No deaths	No deaths	65 ^1^	68 ± 4 ^3^*	67 ± 6 ^2^	67 ^1^	71 ± 11 ^2^
		24–<48 h	51 ± 29 ^4^	57 ± 13 ^4^ n = 1 DNC	No deaths	52 ± 29 ^3^	58 ± 14 ^3^	74 ± 13 ^5^	54 ^1^	No deaths	72 ± 8 ^8^
		48 h	76 ± 9 ^5^	No survivors	78 ± 6	76 ± 9 ^9^	55 ^1^	80 ^1^	77 ± 7 ^8^	80 ± 3 ^3^	No survivors
		48–<72 h	59 ^1^	No survivors	No deaths	No deaths	No deaths	29 ± 33 ^3^	65 ± 6 ^2^	76 ^1^	No survivors
		72 h	82 ± 2 ^5^	No survivors	81 ± 4	82 ± 2 ^9^	81 ^1^	78 ^1^	83 ± 5 ^6^	83 ± 4 ^2^	No survivors
	ML (%)	Baseline	6.5 ± 1.9	6.5 ± 1.9	6.5 ± 1.9	6.5 ± 1.9	6.5 ± 1.9	6.5 ± 1.9	6.5 ± 1.9	6.5 ± 1.9	6.5 ± 1.9
		P1–P2	No deaths	6.5 ± 3.5 ^2^	No deaths	No deaths	4.3 ± 6.7 ^7^	No deaths	DNC	5.5 ± 0.9 ^8^*	5.0 ± 2.8 ^2^*
		P3	3.3 ± 7.1	1.5 ± 2.6 ^8^*	1.2 ± 1.2 *	1.9 ± 0.9	2.6 ± 2.6 ^5^	2.3 ± 3.8	1.2 ± 0.8 ^9^	3.7 ± 4.7 ^3^	1.2 ± 1.0 ^10^*
		P3–<24 h	No deaths	9.3 ± 16.2 ^3^	No deaths	No deaths	1 ^1^	19.7 ± 17.1 ^3^	0.5 ± 0.7 ^2^	0 ^1^	1.0 ± 1.4 ^2^
		24–<48 h	1.0 ± 14 ^4^	0.5 ± 1.0 ^4^ n = 1 DNC	No deaths	2.3 ± 4.0 ^3^	0 ± 0 ^3^	2.8 ± 4.7 ^5^	0 ^1^	No deaths	0.1 ± 0.4 ^8^*
		48 h	1.6 ± 1.1 ^5^*	No survivors	1.2 ± 1.3 *	1.2 ± 1.5 ^9^*	0 ^1^	1 ^1^	1.1 ± 1.1 ^8^*	0.7 ± 1.2 ^3^*	No survivors
		48–<72 h	0 ^1^	No survivors	No deaths	No deaths	No deaths	0 ± 0 ^3^	0 ± 0 ^2^	0 ^1^	No survivors
		72 h	0.3 ± 0.5 ^5¶¶^	No survivors	1.6 ± 1.5 *	1.8 ± 1.4 ^9^*	0 ^1^	0 ^1^	0.8 ± 1.0 ^6^*	1.0 ± 0 ^2^	No survivors
INTEM	CT (s)	Baseline	100 ± 28	100 ± 28	100 ± 28	100 ± 28	100 ± 28	100 ± 28	100 ± 28	100 ± 28	100 ± 28
		P1–P2	No deaths	381 ^1^ n = 1 DNC	No deaths	No deaths	148 ± 86 ^7^	No deaths	DNC	189 ± 142 ^8^	198 ± 5 ^2^
		P3	1332 ± 1205 ^4^ n = 6 DNC	41 ^1^ n = 7 DNC	1391 ± 1228 ^2^* n = 8 DNC	137 ± 29 *	130 ± 19 ^9^	172 ± 38^§^	149 ± 28 ^9^*	140 ± 28 ^3^	172 ± 34*
		P3–<24 h	No deaths	309 ^1^ n = 2 DNC	No deaths	No deaths	129 ^1^	128 ± 28 ^3^	174 ± 2 ^2^*	129 ^1^	146 ± 41 ^3^
		24–<48 h	154 ± 85 ^2^ n = 2 DNC	468 ^1^ n = 4 DNC	No deaths	275 ± 153 ^3^	291 ± 20 ^3††^	183 ± 53 ^5^*	254 ^1^	No deaths	184 ± 24 ^8^*
		48 h	2210 ± 837 ^3^* n = 2 DNC	No survivors	765 ± 498 ^3^ n = 4 DNC	165 ± 72 ^9^*	211 ^1^	152 ^1^	170 ± 39 ^8^*	159 ± 14 ^3^*	No survivors
		48–<72 h	2977 ^1^	No survivors	No deaths	No deaths	No deaths	431 ^1^ n = 1 DNC	218 ± 7 ^2^	224 ^1^	No survivors
		72 h	351 ± 103 ^4^* n = 1 DNC	No survivors	255 ± 181	124 ± 11 ^9^*	187 ^1^	226 ^1^	161 ± 13 ^6^***	144 ± 14 ^2^	No survivors
	MCF (mm)	Baseline	77 ± 1	77 ± 1	77 ± 1	77 ± 1	77 ± 1	77 ± 1	77 ± 1	77 ± 1	77 ± 1
		P1–P2	No deaths	68 n = 1 DNC	No deaths	No deaths	56 ± 22 ^7^	No deaths	DNC	52 ± 25 ^8^	57 ± 2 ^2^
		P3	19 ± 17 ^4¶^ n = 6 DNC	20 ^1^ n = 7 DNC	9 ± 5 ^2^* n = 8 DNC	73 ± 3 *	69 ± 3 ^9§^	70 ± 3^§^	72 ± 2 ^9^*	65 ± 14 ^3^	68 ± 4 ^10^**
		P3–<24 h	No deaths	38 ^1^ n = 2 DNC	No deaths	No deaths	62 ^1^	65 ± 8 ^3^	63 ± 9 ^2^	69 ^1^	69 ± 13 ^3^
		24–<48 h	49 ± 16 ^2^ n = 2 DNC	29 ^1^ n = 4 DNC	No deaths	52 ± 29 ^3^	60 ± 11 ^3^	71 ± 11 ^5^	50 ^1^	No deaths	70 ± 9 ^8^
		48 h	5 ± 1 ^3¶^ n = 2 DNC	No survivors	40 ± 38 ^3^ n = 4 DNC	69 ± 20 ^9^	53 ^1^	77 ^1^	75 ± 7 ^8^	78 ± 3 ^3^	No survivors
		48–<72 h	4 ^1^	No survivors	No deaths	No deaths	No deaths	27 ^1^ n = 1 DNC	66 ± 6 ^2^	75 ^1^	No survivors
		72 h	55 ± 13 ^4^ n = 1 DNC	No survivors	75 ± 22	81 ± 2 ^9^	79 ^1^	77 ^1^	81 ± 6 ^6^	65 ± 24 ^2^	No survivors
	ML (%)	Baseline	6.5 ± 1.9	6.5 ± 1.9	6.5 ± 1.9	6.5 ± 1.9	6.5 ± 1.9	6.5 ± 1.9	6.5 ± 1.9	6.5 ± 1.9	6.5 ± 1.9
		P1–P2	No deaths	0n = 1 DNC	No deaths	No deaths	6.3 ± 8.5 ^7^	No deaths	DNC	3.9 ± 5.2 ^8^	36.5 ± 12.0 ^2^
		P3	25 ± 50 ^4^ n = 6 DNC	0 ^1^ n = 7 DNC	0 ± 0 ^2^ n = 8 DNC	2.2 ± 1.0	1.3 ± 1.0 ^9^	1.1 ± 0.8	1.4 ± 1.1 ^9^	1.0 ± 1.7 ^3^	1.2 ± 0.9 ^10^
		P3–<24 h	No deaths	7 ^1^ n = 2 DNC	No deaths	No deaths	0 ^1^	14.3 ± 12.9 ^3^	0.5 ± 0.7 ^2^	0 ^1^	1.5 ± 2.1 ^3^
		24–<48 h	0.5 ± 0.7 ^2^ n = 2 DNC	0 ^1^ n = 4 DNC	No deaths	0 ± 0 ^3^	0 ± 0 ^3^	1.8 ± 4.0 ^5^	0 ^1^	No deaths	0 ± 0 ^8^*
		48 h	57 ± 49.9 ^3^ n = 2 DNC	No survivors	0.3 ± 0.6 ^3^* n = 4 DNC	1.2 ± 1.1 ^9^*	0 ^1^	1 ^1^	1.4 ± 1.5 ^8^*	1.0 ± 1.0 ^3^*	No survivors
		48–<72 h	78 ^1^	No survivors	No deaths	No deaths	No deaths	0 ^1^ n = 1 DNC	0 ± 0 ^2^	0 ^1^	No survivors
		72 h	0 ± 0 ^4^* n = 1 DNC	No survivors	0.8 ± 1.1 *	1.4 ± 1.4 ^9^	0 ^1^	0 ^1^	1.2 ± 1.5 ^6^	1.0 ± 1.4 ^2^	No survivors
FIBTEM	CT (s)	Baseline	37 ± 3	37 ± 3	37 ± 3	37 ± 3	37 ± 3	37 ± 3	37 ± 3	37 ± 3	37 ± 3
		P1–P2	No deaths	47 ± 1 ^2^	No deaths	No deaths	390 ± 820 ^7^*	No deaths	1862 ^1^	157 ± 272 ^8^*	65 n = 1 DNC
		P3	75 ± 108	48 ± 13 ^7^ n = 1 DNC	45 ± 6 ^2^ n = 1 DNC	41 ± 6	42 ± 5 ^5^	48 ± 6	41 ± 4 ^9^	37 ± 2 ^3^	54 ± 5 ^10^
		P3–<24 h	No deaths	324 ± 368 ^3^	No deaths	No deaths	43 ^1^	47 ± 16 ^3^	58 ± 3 ^2^	40 ^1^	59 ± 7 ^2^
		24–<48 h	45 ± 11 ^4^	37 ± 24 ^2^ n = 2 DNC	No deaths	77 ± 26 ^3‡‡^	48 ± 0 ^3^*	49 ± 5 ^5^*	41 ^1^	No deaths	59 ± 13 ^8^*
		48 h	39 ± 6 ^5^	No survivors	42 ± 5	39 ± 3 ^9^	44 ^1^	42 ^1^	46 ± 10 ^8^	45 ± 7 ^3^	No survivors
		48–<72 h	32 ^1^	No survivors	No deaths	No deaths	No deaths	48 ^1^ n = 1 DNC	41 ± 4 ^2^	58 ^1^	No survivors
		72 h	30 ± 20 ^5^	No survivors	39 ± 3	38 ± 4 ^9^	43 ^1^	40 ^1^	42 ± 2 ^6^	41 ± 9 ^2^	No survivors
	MCF (mm)	Baseline	18 ± 2	18 ± 2	18 ± 2	18 ± 2	18 ± 2	18 ± 2	18 ± 2	18 ± 2	18 ± 2
		P1–P2	No deaths	12 ± 0 ^2^*	No deaths	No deaths	10 ± 6 ^7^*	No deaths	4 ^1^	10 ± 4 ^8^*	11 n = 1 DNC
		P3	21 ± 13	15 ± 3 ^7^ n = 1 DNC	16 ± 3 ^2^ n = 1 DNC	20 ± 3	16 ± 2 ^5^	21 ± 16	15 ± 2 ^9^	16 ± 2 ^3^	12 ± 3 ^10^
		P3–<24 h	No deaths	14 ± 15 ^3^	No deaths	No deaths	10 ^1^	18 ± 10 ^3^	10 ± 1 ^2^	11 ^1^	16 ± 13 ^2^
		24–<48 h	33 ± 13 ^4^	37 ± 10 ^2^ n = 2 DNC	No deaths	36 ± 14 ^3^	44 ± 6 ^3^	35 ± 9 ^5^	50 ^1^	No deaths	35 ± 15 ^8^
		48 h	50 ± 23 ^5^*	No survivors	45 ± 7 *	48 ± 7 ^9^*	44 ^1^	52 ^1^	45 ± 7 ^8^*	46 ± 3 ^3^*	No survivors
		48–<72 h	54 ^1^	No survivors	No deaths	No deaths	No deaths	26 ^1^ n = 1 DNC	48 ± 8 ^2^	45 ^1^	No survivors
		72 h	50 ± 5 ^5^*	No survivors	44 ± 8 *	45 ± 8 ^9^*	48 ^1^	45 ^1^	50 ± 8 ^6^*	49 ± 1 ^2^*	No survivors
	ML (%)	Baseline	2.0 ± 3.2	2.0 ± 3.2	2.0 ± 3.2	2.0 ± 3.2	2.0 ± 3.2	2.0 ± 3.2	2.0 ± 3.2	2.0 ± 3.2	2.0 ± 3.2
		P1–P2	No deaths	0 ± 0 ^2^	No deaths	No deaths	16 ± 37 ^7^	No deaths	72 ^1^	8 ± 18 ^8^	0n = 1 DNC
		P3	0.7 ± 2.2	0.4 ± 1.1 ^7^ n = 1 DNC	0 ± 0 ^2^ n = 1 DNC	0 ± 0	0.2 ± 0.4 ^5^	0.1 ± 0.3	0 ± 0 ^9^	0 ± 0 ^3^	0.7 ± 2.2 ^10^
		P3–<24 h	No deaths	14.3 ± 24.8 ^3^	No deaths	No deaths	1 ^1^	0 ± 0 ^3^	0 ± 0 ^2^	0 ^1^	0 ± 0 ^2^
		24–<48 h	0 ± 0 ^4^	0 ± 0 ^2^ n = 2 DNC	No deaths	0 ± 0 ^3^	0 ± 0 ^3^	0 ± 0 ^5^	0 ^1^	No deaths	0.1 ± 0.4 ^8^
		48 h	0 ± 0 ^5^	No survivors	0.5 ± 1.6	0 ± 0 ^9^	0 ^1^	0 ^1^	0 ± 0 ^8^	0 ± 0 ^3^	No survivors
		48–<72 h	0 ^1^	No survivors	No deaths	No deaths	No deaths	0 ^1^ n = 1 DNC	0 ± 0 ^2^	0 ^1^	No survivors
		72 h	0 ± 0 ^5^*	No survivors	1.4 ± 4.4	0.3± 1.0 ^9^*	0 ^1^	0 ^1^	0 ± 0 ^6^	0 ± 0 ^2^	No survivors
FIBTEM MCF: EXTEM MCF		Baseline	0.23 ± 0.02	0.23 ± 0.02	0.23 ± 0.02	0.23 ± 0.02	0.23 ± 0.02	0.23 ± 0.02	0.23 ± 0.02	0.23 ± 0.02	0.23 ± 0.02
		P1–P2	No deaths	0.17 ± 0.00 ^2^	No deaths	No deaths	0.16 ± 0.07 ^7^	No deaths	0.17 ^1^	0.17 ± 0.03 ^7^	0.17 ^1^
		P3	0.29 ± 0.18 ^9^	0.22 ± 0.04 ^7^	0.22 ± 0.04 ^9^	0.27 ± 0.05	0.23 ± 0.02 ^5^	0.29 ± 0.23	0.21 ± 0.02 ^9^	0.22 ± 0.02 ^3^	0.17 ± 0.04 ^10^
		P3–<24 h	No deaths	0.60 ± 0.48 ^3^	No deaths	No deaths	0.15 ^1^	0.26 ± 0.07 ^3^	0.14 ± 0.02 ^2^	0.16 ^1^	0.21 ± 0.16 ^2^
		24–<48 h	0.72 ± 0.23 ^4*^	0.67 ± 0.19 ^4^*	No deaths	0.79 ± 0.31 ^3^*	0.77 ± 0.09 ^3†††^	0.45 ± 0.06 ^5^*	0.93 ^1^	No deaths	0.48 ± 0.18 ^8^*
		48 h	0.68 ± 0.14 ^5^*	No survivors	0.57 ± 0.07 *	0.64 ± 0.11 ^9^*	0.65 ^1^	0.61 ^1^	0.60 ± 0.12 ^8^*	0.58 ± 0.05 ^3^*	No survivors
		48–<72 h	0.92 ^1^	No survivors	No deaths	No deaths	No deaths	0.67 ± 0.38 ^2^	0.74 ± 0.18 ^2^	0.59 ^1^	No survivors
		72 h	0.61 ± 0.04 ^4^*	No survivors	0.54 ± 0.08 *	0.54 ± 0.09 ^9^*	0.59 ^1^	0.58 ^1^	0.60 ± 0.07 ^6^*	0.59 ± 0.03 ^2^*	No survivors

Measurements were taken at end of Phase 3, 48 h, and 72 h (survivors), or at time of sacrifice according to humane endpoints (non-survivors) (see Section 2.6). Data represent mean ± SD. Results for P1–P2, P3–<24 h, 24–<48 h, and 48–<72 h indicated by grey shading are for moribund animals sacrificed prior to experimental end (72 h), i.e., non-survivors. n values for NT, FFP, and FWB groups are n = 10, n = 12, and n = 12, respectively, or indicated by superscript number. Baseline values taken from n = 6 healthy male Sprague-Dawley rats. P1, Phase 1 (60 min bolus resuscitation); P2, Phase 2 (4 h drip resuscitation); P3, Phase 3 (60 min transfusion); ALM, adenosine, lidocaine, magnesium; NT, no transfusion; FFP, fresh frozen plasma; FWB, fresh whole blood; CT, clot time; MCF, maximum clot firmness; ML, maximum lysis; DNC, did not clot. * *p* < 0.05 compared to baseline; ^‡^ *p* < 0.05 compared to baseline, Sham FWB, and Saline FWB; ^§^ *p* < 0.05 compared to baseline and Sham FFP; ^¶^ *p* < 0.05 compared to baseline, Sham FFP, and Sham FWB; ** *p* < 0.05 compared to baseline and Sham FWB; ^††^ *p* < 0.05 compared to baseline, ALM FFP, and ALM FWB; ^†††^ *p* < 0.05 compared to baseline and ALM FFP; ^‡‡^ *p* < 0.05 compared to ALM FFP; ^¶¶^ *p* < 0.05 compared to baseline and ALM NT; *** *p* < 0.05 compared to Sham FFP.

## Data Availability

The raw data supporting the conclusions of this article will be made available by the authors on request.

## Supplementary Materials

The following supporting information can be downloaded at: https://www.mdpi.com/article/10.3390/medicina62030453/s1, Supplementary Material S1: ARRIVE Checklist; Supplementary Figure S1: Hemodynamics; Supplementary Table S1: Blood Chemistry.

## Abbreviations

The following abbreviations are used in this manuscript:

ALM	Adenosine, lidocaine, magnesium
FFP	Fresh frozen plasma
FWB	Fresh whole blood
NT	No transfusion
MAP	Mean arterial pressure
Ampk	5′ adenosine monophosphate-activated protein kinase
Sirt1	Sirtuin 1
Pgc1a	Peroxisome proliferator-activated receptor gamma coactivator 1-alpha
MtCo3	Mitochondrially encoded cytochrome C oxidase III
Tfam	Transcription factor A, mitochondrial
ACURO	Animal Care and Use Review Office
ARRIVE	Animal Research: Reporting In Vivo Experiments
s.c.	Subcutaneous
IV	Intravenous
NaCl	Sodium chloride
MgSO_4_	Magnesium sulfate
PT	Prothrombin time
aPTT	Activated partial thromboplastin time
TAFI	Tissue activatable fibrinolysis inhibitor
PRP	Platelet-rich plasma
ADP	Adenosine diphosphate
H&E	Hematoxylin and eosin
SD	Standard deviation
ANOVA	Analysis of variance
HR	Heart rate
K^+^	Potassium
RBC	Red blood cell
Hct	Hematocrit
Hb	Hemoglobin
WBC	White blood cell
CT	Clot time
MCF	Maximum clot firmness
TRALI	Transfusion-related lung injury
ARDS	Acute respiratory distress syndrome
TIC	Trauma-induced coagulopathy
GPIIb/IIIa	Glycoprotein IIb/IIIa
TRIM	Transfusion-associated immunomodulation
