# Supplementary material for: ALM Resuscitation Without Transfusion Improves Platelet Function and Survival After Liver Injury and Uncontrolled Hemorrhage
Source: Medicina (Kaunas). 2026 Feb 27;62(3):453. doi: 10.3390/medicina62030453 (PMC13027957; doi:10.3390/medicina62030453)
Supplement: Supplementary file 1 [file medicina-62-00453-s001.zip › Supplementary Figure S1 Hemodynamics.pdf]

Supplementary Figure S1

A

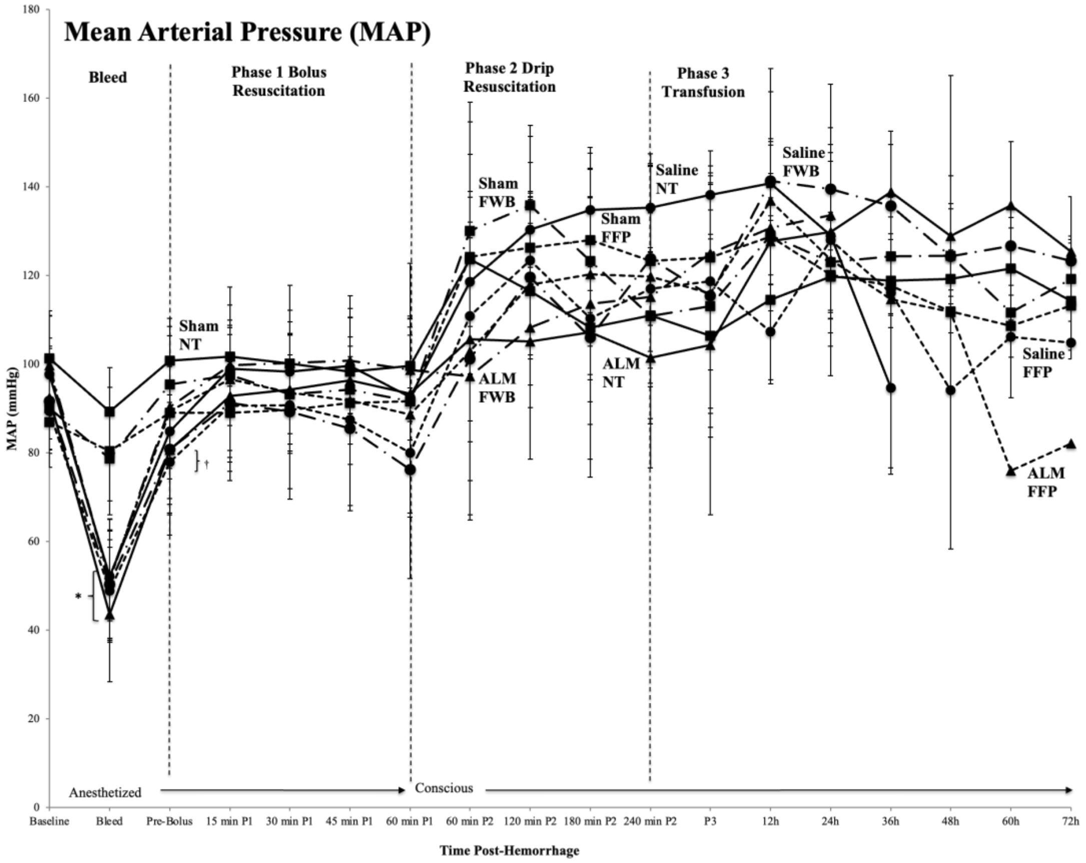

B

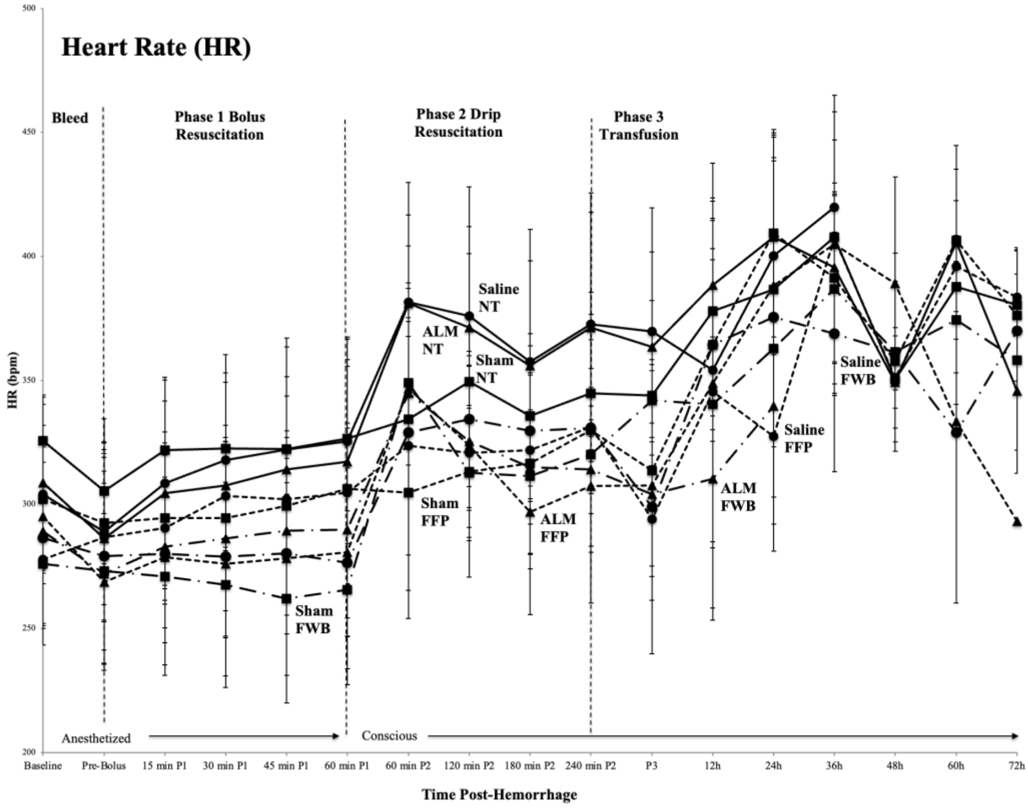

Supplementary Figure S1

Mean arterial pressure (MAP, mmHg) (A) and heart rate (HR, bpm) (B) at baseline, after uncontrolled hemorrhage, during phase 1 (P1), phase 2 (P2) resuscitation, and phase 3 (P3) transfusion, and 72h monitoring Shams (■), Saline controls (●) and ALM treatment group (▲) receiving no transfusion (solid line), FFP (dotted line), or FWB (dashed line). Values represent mean ± SD. * $p<0.05$ compared to Sham NT, Sham FFP, and Sham FWB; † $p<0.05$ compared to Sham NT. ALM, adenosine, lidocaine, magnesium; NT, no transfusion; FFP, fresh frozen plasma; FWB, fresh whole blood.
