# Supplementary material for: ALM Resuscitation Without Transfusion Improves Platelet Function and Survival After Liver Injury and Uncontrolled Hemorrhage
Source: Medicina (Kaunas). 2026 Feb 27;62(3):453. doi: 10.3390/medicina62030453 (PMC13027957; doi:10.3390/medicina62030453)
Supplement: Supplementary file 1 [file medicina-62-00453-s001.zip › Supplementary Table S1.pdf]

SUPPLEMENTARY TABLE S1: Blood Chemistry for Shams, Saline controls, and ALM group without transfusion, and with FFP or FWB following laparotomy, liver resection and hemorrhage. Measurements were taken at baseline, end of Phase 3, and 72h (survivors), or at time of sacrifice according to humane endpoints (non-survivors) (see Methods).

Parameter	Time	Sham NT	Saline NT	ALM NT	Sham FFP	Saline FFP	ALM FFP	Sham FWB	Saline FWB	ALM FWB
**K⁺** (mM) [3.5 – 5.0]	Baseline	4.65 ± 0.51[10]	4.49 ± 0.33[10]	4.37 ± 0.35[10]	4.25 ± 0.50[12]	4.36 ± 0.39[12]	4.26 ± 0.55[12]	4.36 ± 0.32[12]	4.63 ± 0.37[12]	4.21 ± 0.41[12]
	P1-P2	No deaths	6.40 ± 1.41[2]	No deaths	No deaths	6.94 ± 2.01[7*]	No deaths	12.3[1]	8.79 ± 4.90[8]	5.55 ± 0.35[2]
	P3	4.01 ± 0.28[10*]	4.09 ± 0.34[8]	4.25 ± 0.16[10]	3.86 ± 0.28[12†]	4.26 ± 0.79[5]	4.33 ± 0.64[12]	4.36 ± 0.87[11]	3.95 ± 0.26[4*]	4.13 ± 0.44[10]
	P3-<24h	No deaths	14.25 ± 3.18[2]	No deaths	No deaths	5.55[1]	8.07 ± 1.91[3]	7.30 ± 4.24[2]	4.20[1]	5.55 ± 1.34[2]
	24-<48h	11.88 ± 6.55[4]	6.85 ± 2.70[5]	No deaths	7.57 ± 1.70[3]	8.77 ± 6.23[3]	5.54 ± 1.67[5]	7.6[1]	No deaths	5.50 ± 1.10[8‡]
	48-<72h	No Sample	No Survivors	No deaths	No deaths	No deaths	7.20 ± 2.62[3]	5.60 ± 1.41[2]	4.80[1]	No Survivors
	72h	4.20 ± 0.31[5*]	No Survivors	4.02 ± 0.40[10]	4.57 ± 0.28[9]	4.40[1]	4.50[1]	4.25 ± 0.36[6]	4.10 ± 0.14[2]	No Survivors
**Na⁺** (mM) [126 – 145]	Baseline	131 ± 1[10§]	133 ± 1[10]	130 ± 1[10‖]	137 ± 7[12]	134 ± 2[12]	139 ± 12[12]	139 ± 2[12]	137 ± 5[12]	136 ± 8[12]
	P3	No deaths	137 ± 2[2]	No deaths	No deaths	157 ± 15[7*]	No deaths	140[1]	147 ± 18[8]	135 ± 16[2]
	Phase 3	130 ± 6[10]	134 ± 4[8]	133 ± 5[10]	153 ± 13[12¶]	168 ± 32[5]	137 ± 10[12]	153 ± 15[11**]	159 ± 17[4]	134 ± 10[10]
	P3-<24h	No deaths	132 ± 4[2]	No deaths	No deaths	126[1]	126 ± 7[3]	136 ± 0[2]	122[1]	134 ± 11[2]
	24-<48h	131 ± 11[4]	139 ± 1[2]	No deaths	145 ± 5[3]	146 ± 19[3]	134 ± 4[5]	139[1]	No deaths	135 ± 4[8]
	48-<72h	No Sample	No Survivors	No deaths	No deaths	No deaths	132 ± 5[3]	147 ± 11	122[1]	No Survivors
	72h	140 ± 2[5*]	No Survivors	145 ± 9[10*]	166 ± 15[9††]	141[1]	146[1]	158 ± 15[6‡‡]	154 ± 10[2]	No Survivors
**Ca²⁺** (mM) [1.10 – 1.44]	Baseline	1.33 ± 0.03[10§§]	1.34 ± 0.02[10§§]	1.30 ± 0.06[10§§]	1.41 ± 0.04[12]	1.42 ± 0.05[12]	1.43 ± 0.15[12]	1.40 ± 0.03[12]	1.41 ± 0.04[12]	1.40 ± 0.09[12]
	P1-P2	No deaths	1.24 ± 0.01[2]	No deaths	No deaths	1.38 ± 0.07[7]	No deaths	1.46[1]	1.41 ± 0.12[8]	1.42 ± 0.03[2]
	P3	1.17 ± 0.05[10*]	1.16 ± 0.03[8*]	1.16 ± 0.04[10†]	1.26 ± 0.05[12†]	1.22 ± 0.03[12*]	1.17 ± 0.17[12*]	1.24 ± 0.05[11*]	1.26 ± 0.02[4*]	1.19 ± 0.04[10*]
	P3-<24h	No deaths	1.11 ± 0.04[3]	No deaths	No deaths	1.17[1]	1.04 ± 0.21[3]	1.26 ± 0.13[2]	1.28[1]	1.16 ± 0.12[2]
	24-<48h	1.12 ± 0.04[4*]	1.14 ± 0.04[4*]	No deaths	1.08 ± 0.12[3]	1.27 ± 0.08[3]	1.07 ± 0.22[5]	1.14[1]	No deaths	1.11 ± 0.10[8‡]
	48-<72h	No Sample	No Survivors	No deaths	No deaths	No deaths	1.17 ± 0.12[3]	1.30 ± 0.09[2]	1.50[1]	No Survivors
	72h	1.25 ± 0.03[5‖‖]	No Survivors	1.31 ± 0.03[10]	1.35 ± 0.05[9*]	1.34[1]	1.35[1]	1.30 ± 0.07[6]	1.37 ± 0.02[2]	No Survivors
**Cl⁻** (mM) [95 – 110]	Baseline	105 ± 2[10]	109 ± 2[10]	109 ± 2[10]	103 ± 6[12¶¶]	101 ± 1[12***]	103 ± 5[12¶¶]	107 ± 3[12]	107 ± 6[12]	107 ± 4[12]
	P1-P2	No deaths	115 ± 4[2]	No deaths	No deaths	125 ± 13[7*]	No deaths	105[1]	118 ± 16[8]	118 ± 0[2]
	P3	101 ± 5[10]	108 ± 6[8]	109 ± 7[9]	115 ± 12[12*]	135 ± 36[5]	99 ± 5[12†††]	121 ± 17[11*]	129 ± 18[4]	102 ± 2[10*]
	P3-<24h	No deaths	109 ± 10[2]	No deaths	No deaths	92[1]	102 ± 4[3]	110 ± 6[2]	154[1]	101 ± 9[2]
	24-<48h	104 ± 9[4]	111 ± 5[2]	No deaths	108 ± 6[3]	112 ± 17[3]	100 ± 4[5*]	110[1]	No deaths	103 ± 2[8*]
	48-<72h	No Sample	No Survivors	No deaths	No deaths	No deaths	102 ± 9[3]	111 ± 12[2]	134[1]	No Survivors
	72h	110 ± 2[5*]	No Survivors	119 ± 9[10*]	130 ± 14[9‡]	107[1]	109[1]	124 ± 17[6]	122 ± 7[2]	No Survivors
**Base Excess** (mM)	Baseline	2.6 ± 1.3[10]	3.4 ± 1.9[10]	3.5 ± 1.5[10]	2.2 ± 1.1[12]	2.9 ± 1.7[12]	4.1 ± 2.3[12]	2.5 ± 1.8[12]	3.3 ± 1.9[12]	5.3 ± 3.3[12]
	P1-P2	No deaths	-13.7 ± 2.6[2]	No deaths	No deaths	-15.4 ± 9.2[7*]	No deaths	-15.9[1]	-13.5 ± 5.6[8]	-22.2 ± 0.1[2]
	P3	2.8 ± 4.7[10]	3.5 ± 2.3[8]	4.1 ± 3.3[10]	5.7 ± 2.3[12†]	-0.2 ± 6.0[5]	4.5 ± 10.3[12]	0.1 ± 9.1[11]	-1.9 ± 10.8[4]	10.4 ± 3.7[10‡‡‡]
	P3-<24h	No deaths	-14.1 ± 0.5[2*]	No deaths	No deaths	-5.0[1]	-14.1 ± 6.6[3]	-17.7 ± 6.8[2]	-17.3[1]	3.4 ± 10.7[2]
	24-<48h	-10.1 ± 7.1[4*]	-7.1 ± 5.4[4]	No deaths	-7.5 ± 5.2[3]	-4.6 ± 10.6[3]	5.0 ± 8.4[5§§§]	0.0[1]	No deaths	8.5 ± 4.5[8‖‖‖]
	48-<72h	No Sample	No Survivors	No deaths	No deaths	No deaths	-4.2 ± 12.8[3]	-1.2 ± 7.4[2]	0.2[1]	No Survivors
	72h	2.6 ± 0.9[5]	No Survivors	4.4 ± 1.2[10]	2.7 ± 3.0[9]	3.8[1]	0.7[1]	2.6 ± 3.0[6]	3.9 ± 1.8[2]	No Survivors

**HCO_3^-** §§§§ (mM)	P1-P2	No deaths	13.0^{1}	No deaths	No deaths	11.5 ± 5.9^{6}	No deaths	6.9^{1}	11.7 ± 4.8^{8}	8.0 ± 0.5^{2}
	P3	29.9 ± 1.7^{5}	27.6 ± 2.1^{8}	28.9 ± 2.5^{9}	30.0 ± 1.5^{11}	24.5 ± 5.0^{5}	28.6 ± 8.2^{12}	25.7 ± 6.8^{9}¶¶¶	23.3 ± 8.5^{4}¶¶¶	33.2 ± 3.4^{10}
	P3-<24h	No deaths	11.7 ± 1.0^{3}	No deaths	No deaths	20.1^{1}	10.7 ± 3.5^{2}	6.2^{1}	No sample	27.8 ± 9.7^{2}
	24-<48h	15.2 ± 6.5^{4}	19.1 ± 4.5^{5}****	No deaths	14.3 ± 2.2^{3}	19.5 ± 14.8^{2}	29.2 ± 7.6^{5}	No sample	No deaths	31.6 ± 4.2^{7}††††
	48-<72h	No Sample	No Survivors	No deaths	No deaths	No deaths	46.6 ± 29.1^{3}	68.1 ± 23.3^{2}	39.8^{1}	No Survivors
	72hr	27.3 ± 0.9^{5}****	No Survivors	28.7 ± 2.7^{8}	27.7 ± 2.7^{8}	28.1^{1}	25.0^{1}	27.1 ± 2.8^{4}	28.4 ± 1.4^{2}	No Survivors

Data represent mean±SD. Normal ranges for healthy Male Sprague-Dawley rats from James Cook University Breeding Colony indicated in square parentheses in Parameter column. Results for P1-P3, P3-24h, 24-48h, and 48-72h highlighted in grey shading, are for moribund animals sacrificed prior to experimental end (72h), i.e. non-survivors. n value indicated by superscript number. P1, Phase 1 (60 min bolus resuscitation); P2, Phase 2 (4h drip resuscitation); P3, Phase 3 (60 min transfusion); ALM, adenosine, lidocaine, magnesium; NT, no transfusion; FFP, fresh frozen plasma; FWB, fresh whole blood; K^+, potassium; Na^+, sodium; Ca^{2+}, calcium; Cl^-, chloride; HCO_3^-, bicarbonate. *$p<0.05$ compared to Baseline; †$p<0.05$ compared to Baseline and 72h; ‡$p<0.05$ compared to Baseline and Phase 3; §$p<0.05$ compared to Saline FFP, Sham FWB, and Saline FWB; ‖$p<0.05$ compared to Saline NT, Saline FFP, Sham FWB, and Saline FWB; ¶$p<0.05$ compared to Sham NT, Saline NT, ALM NT, ALM FWB, and Baseline; **$p<0.05$ compared to Sham NT and Baseline; ††$p<0.05$ compared to Sham NT, ALM NT, and Baseline; ‡‡$p<0.05$ compared to Sham NT; §§$p<0.05$ compared to Sham FFP, Saline FFP, Sham FWB, and Saline FWB; ‖‖$p<0.05$ compared to ALM NT, Sham FFP, Saline FWB, and Baseline; ¶¶$p<0.05$ compared to Saline NT and ALM NT; ***$p<0.05$ compared to Saline NT, ALM NT, Sham FWB, Saline FWB, and ALM FWB; †††$p<0.05$ compared to Sham FFP; ‡‡‡$p<0.05$ compared to Sham NT, Saline NT, Saline FFP, Sham FFP, and Baseline; §§§$p<0.05$ compared to Sham NT and Saline NT; ‖‖‖$p<0.05$ compared to Sham NT, Saline NT, Sham FFP, and Saline FFP; ¶¶¶$p<0.05$ compared to ALM FWB; ****$p<0.05$ compared to Phase 3; ††††$p<0.05$ compared to Saline NT and Sham FFP. §§§§ No baseline measurements and reduced sample numbers for HCO_3^- due to effect of anesthesia or oximetry measuring errors.
